# Hepatic Ago2-mediated RNA silencing controls energy metabolism linked to AMPK activation and obesity-associated pathophysiology

**DOI:** 10.1038/s41467-018-05870-6

**Published:** 2018-09-10

**Authors:** Cai Zhang, Joonbae Seo, Kazutoshi Murakami, Esam S. B. Salem, Elise Bernhard, Vishnupriya J. Borra, Kwangmin Choi, Celvie L. Yuan, Calvin C. Chan, Xiaoting Chen, Taosheng Huang, Matthew T. Weirauch, Senad Divanovic, Nathan R. Qi, Hala Einakat Thomas, Carol A. Mercer, Haruhiko Siomi, Takahisa Nakamura

**Affiliations:** 1 Division of Endocrinology, Cincinnati Children’s Hospital Medical Center, Cincinnati, OH USA; 2 Department of Pediatrics, Tongji Hospital, Tongji Medical College, Huazhong University of Science and Technology, Wuhan, China; 3 Department of Pharmacology and Systems Physiology, University of Cincinnati College of Medicine, Cincinnati, OH USA; 4 Division of Experimental Hematology and Cancer Biology, Cincinnati Children’s Hospital Medical Center, Cincinnati, OH USA; 5 Medical Scientist Training Program, Immunology Graduate Program, Cincinnati Children’s Hospital Medical Center and University of Cincinnati College of Medicine, Cincinnati, OH USA; 6 Division of Biomedical Informatics, Cincinnati Children’s Hospital Medical Center, Cincinnati, OH USA; 7 Department of Pediatrics, University of Cincinnati College of Medicine, Cincinnati, OH USA; 8 Division of Human Genetics, Cincinnati Children’s Hospital Medical Center, Cincinnati, OH USA; 9 Division of Developmental Biology, Cincinnati Children’s Hospital Medical Center, Cincinnati, OH USA; 10 Center for Autoimmune Genomics and Etiology, Cincinnati Children’s Hospital Medical Center, Cincinnati, OH USA; 11 Division of Immunobiology, Cincinnati Children’s Hospital Medical Center, Cincinnati, OH USA; 12 Department of Internal Medicine, University of Michigan Medical School, Ann Arbor, MI USA; 13 Division of Hematology-Oncology, Department of Internal Medicine, University of Cincinnati College of Medicine, Cincinnati, OH USA; 14 Department of Molecular Biology, Keio University School of Medicine, Tokyo, Japan

## Abstract

RNA silencing inhibits mRNA translation. While mRNA translation accounts for the majority of cellular energy expenditure, it is unclear if RNA silencing regulates energy homeostasis. Here, we report that hepatic Argonaute 2 (Ago2)-mediated RNA silencing regulates both intrinsic energy production and consumption and disturbs energy metabolism in the pathogenesis of obesity. Ago2 regulates expression of specific miRNAs including miR-802, miR-103/107, and miR-148a/152, causing metabolic disruption, while simultaneously suppressing the expression of genes regulating glucose and lipid metabolism, including *Hnf1β*, *Cav1*, and *Ampka1*. Liver-specific Ago2-deletion enhances mitochondrial oxidation and ATP consumption associated with mRNA translation, which results in AMPK activation, and improves obesity-associated pathophysiology. Notably, hepatic Ago2-deficiency improves glucose metabolism in conditions of insulin receptor antagonist treatment, high-fat diet challenge, and hepatic AMPKα1-deletion. The regulation of energy metabolism by Ago2 provides a novel paradigm in which RNA silencing plays an integral role in determining basal metabolic activity in obesity-associated sequelae.

## Abstract (web summary)

The RNA-induced silencing complex (RISC) represses gene expression via micro-RNA guided mRNA silencing. Here, the authors show that RISC component Argonaute 2 in the liver regulates energy metabolism by inducing microRNAs that cause metabolic disruption and by suppressing protein translation linked to AMPK activation.

## Introduction

The worldwide prevalence of obesity has reached pandemic proportions, bringing with it a host of associated diseases, such as type 2 diabetes (T2D) and non-alcoholic steatohepatitis (NASH)^1,2^. Obesity develops when energy intake chronically exceeds total energy expenditure. Basal metabolic rate represents the largest component of total energy expenditure, of which the liver is a major organ for energy consumption^3^. Protein biosynthesis is one of the most energy-consuming cellular processes in the liver, accounting for ~20–30% of total energy consumption^4,5^. However, despite abundant supply of energy sources and a robust activation of the mammalian target of rapamycin complex (mTORC) pathway, a main driver of protein synthesis^6–9^, the liver-driven energy consumption robustly declined in obesity due to, at least in part, insufficient protein biosynthesis. Suppression of hepatic protein synthesis leads to further accumulation of energy sources associated with obesity-associated pathophysiology, however, the exact mechanism(s) of insufficient protein biosynthesis remains unclear. Hence, defining such molecular mechanism(s) could provide a novel therapeutic approach that alters energy balance in obesity and modulates the pathogenesis of associated sequelae.

Recent studies have revealed significant roles for microRNA (miRNA)-mediated events in the development and progression of obesity and its associated sequelae^10,11^. Global dysregulation of miRNA expression is triggered in the human and murine obese liver, leading to the induction of the vast majority of miRNAs, including miR-802, miR-103/miR-107, and miR-148a that deteriorate glucose and lipid metabolism in obesity^12–16^. As miRNA generally inhibits the translation of target mRNAs through RNA silencing, it is reasonable to hypothesize that these induced miRNAs may contribute to suppression of protein biosynthesis and its associated energy expenditure in obese liver. However, there remain fundamental questions concerning why and how these miRNAs are concurrently induced in the obese condition and whether RNA silencing is integrated into an elaborate adaptive program that cells can elicit to balance anabolic and catabolic processes dependent on energy and metabolic statuses. If RNA silencing plays a role in protein biosynthesis-associated energy metabolism, one would anticipate that individual component(s) of miRNA-regulatory machinery in the liver may impinge on metabolic regulation, and that a nutrient challenge might accentuate the consequences of this regulation.

Argonaute (Ago) family proteins are the main components of the RNA-induced silencing complex (RISC) that carries out RNA silencing. Upon loading of Ago proteins with mature miRNAs produced by the endoribonuclease Dicer^17,18^, RISC represses the expression of targeted mRNA through RNA silencing^18–20^. Amongst all Ago proteins, Ago2 specifically possesses an endoribonuclease (“slicer”) activity that generates a specific mature miRNA and cleaves targeted mRNAs in mammals^18,21–24^. To study the role of RNA silencing in hepatic energy homeostasis, we comprehensively evaluated the role of hepatic Ago1 and Ago2, as analyses of these core RISC components might lead to fundamental insights into the link of RNA silencing with energy metabolism. This study demonstrates that hepatic Ago2-mediated RNA silencing regulates energy expenditure during the course of obesity and its inactivation protects from obesity-associated glucose intolerance and hepatic steatosis in mice. Importantly, we discover novel roles of Ago2 in orchestrating the expression of a subset of miRNAs, including miR-802, miR-103/107, and miR-148a, and in the regulation of AMP-activated protein kinase (AMPK) activation linked to protein biosynthesis-mediated energy consumption. This Ago2-mediated RNA silencing is a critical mechanism that connects the dots between protein translation, energy production and consumption, and AMPK activity—disruption of such events is a well-recognized feature in obesity and the pathogenesis of obesity-associated sequelae.

## Results

### Hepatic Ago2 regulates expression of specific miRNAs involved in energy metabolism

Ago1 and Ago2 are the predominant Ago family members expressed in the liver^25^. To investigate if RNA silencing is associated with energy and metabolic homeostasis, we generated liver-specific Ago1-deficient (L-Ago1 KO: *Ago1*^*fl/fl*^ *Alb-Cre*^*Tg/0*^) and Ago2-deficient (L-Ago2 KO: *Ago2*^*fl/fl*^ *Alb-Cre*^*Tg/0*^) mice (Supplementary Fig. 1a and b). On control diet (CD), both L-Ago1 KO and L-Ago2 KO mice gained weight similarly to their controls, L-Ago1 WT (*Ago1*^*fl/fl*^ *Alb-Cre*^*0/0*^) or L-Ago2 WT (*Ago2*^*fl/fl*^ *Alb-Cre*^*0/0*^), and showed no obvious abnormalities during development or in adulthood. In addition, levels of serum alanine aminotransferase (ALT) were comparable between the groups (Supplementary Fig. 1c and d). These results suggest that general functions of the liver are likely unaffected by the absence of Ago1 or Ago2 during development in regular feeding conditions.

**Fig. 1:**
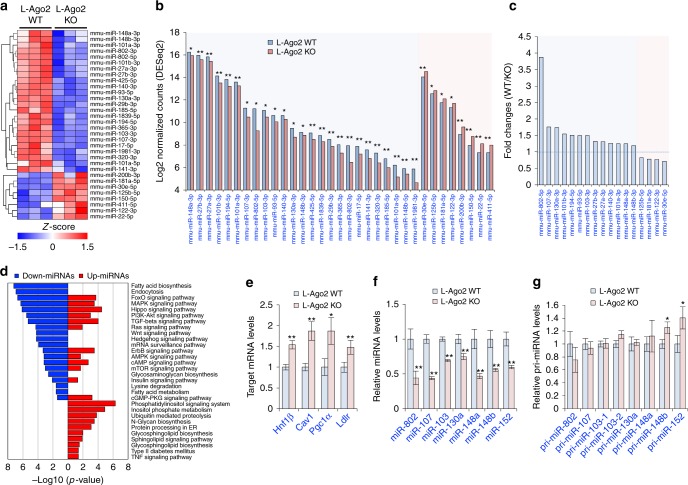
Effects of hepatic Ago2-deficiency on MD-miRNA expression in the liver. **a**–**c** A heatmap diagram illustrating the differential expression of hepatic mature miRNAs (**a**), raw counts of significant miRNAs normalized by DESeq2 and transformed by Log2 (**b**), and fold changes of significant miRNAs whose expression levels are in the top 10 percent in the WT liver (**c**) in the liver of L-Ago2 WT (*n* = 3) and L-Ago2 KO (*n* = 3) mice fed NCD at 9 weeks of age. Significant miRNAs differentially expressed between L-Ago2 WT and L-Ago2 KO groups were identified using DESeq2 (|fold change| > 2x and adjusted *p* < 0.05) and plotted as a heatmap using z-score. **d** Metabolic pathway enrichment analysis of miRNAs significantly downregulated (blue) and upregulated (red) miRNAs in the liver of L-Ago2 KO mice fed NCD at 9 weeks of age. These miRNAs were queried to calculate the most enriched KEGG pathways using DIANA-mirPath web-server (*p* < 0.05 and MicroT threshold < 0.8). Pathways unrelated to hepatic functions were excluded in this pyramid plot. **e**–**g** Expression levels of MD-miRNAs’ target mRNAs (**e**), selective MD-miRNAs (**f**), and their pri-miRNAs (**g**) in the liver of L-Ago2 WT (*n* = 8) and L-Ago2 KO (*n* = 8) mice fed NCD at 25 weeks of age. Data are shown as the mean ± SEM. *p < 0.05, **p < 0.01

Among the Ago proteins, Ago2 uniquely possesses a slicer activity known to contribute to the expression of specific miRNA^21–23^ and mRNA cleavage^18,26^. In the Ago2-deficient livers, expression levels of Ago1 are increased (Supplementary Fig. 1b), and therefore Ago1 may compensate for Ago2’s non-slicer activity-dependent function. To gain insight into the specific roles of Ago2’s activity in the regulation of liver function, we first assessed the effect of hepatic Ago2-deficiency on miRNA expression profile. Expression profile analyses revealed that the expression levels of 25 miRNAs were significantly reduced in L-Ago2 KO liver, while 8 miRNAs were significantly increased (Fig. 1a, b). Among these significant miRNAs, miR-148a is one of the most abundant miRNAs expressing in the WT liver (Fig. 1b and Supplementary Fig. 2a). In addition, 17 out of 33 total significant miRNAs abundantly expressed were in the top 10 percent of miRNAs expressing in the WT liver (Fig. 1c and Supplementary Table 1). Intriguingly, this group contained miRNAs known to be associated with metabolic diseases (MD-miRNAs) and detrimental to glucose and lipid metabolism, including miR-802, miR-103/107, miR-130a, and miR-148a^12,13,27–29^, which were downregulated in the L-Ago2 KO liver (Fig. 1c). We additionally utilized the list of significant miRNAs altered in Ago2 KO liver through the miRNA enrichment pathway analysis by the biological processes and molecular function categories (Fig. 1d). Hepatic Ago2 deficiency affected the functional clades associated with energy metabolism including fatty acid biosynthesis, AMPK signaling, protein processing, and insulin signaling. Taken together, these results imply the unique role of Ago2 in the expressional regulation of a specific repertoire of MD-miRNAs for energy metabolism. Consistent with these observations, the reduction of MD-miRNAs in L-Ago2 KO liver is accompanied by increased expression of their known target mRNAs, such as hepatocyte nuclear factor 1 homeobox B (*Hnf1β*), caveolin-1 (*Cav1*), peroxisome proliferator-activated receptor gamma coactivator 1α (*Pgc1α*), and low-density lipoprotein receptor (*Ldlr*), which regulates energy metabolism (Fig. 1e)^12,13,29^.

To examine the mechanism by which hepatic Ago2 regulates expression of specific miRNAs, we measured the expression levels of each mature and primary miRNA (pri-miRNA) employing TaqMan probe-based gene expression analysis. Mature miRNA levels of miR-802, miR-107/miR-103, miR-130a, and miR-148a/148b/152, were reduced in L-Ago2 KO liver (Fig. 1f), despite intact expression levels of their pri-miRNAs (Fig. 1g). These results suggest that the miRNA maturation process is impaired in the Ago2-deficient liver. To further confirm this regulation, we utilized Ago2-deficient mouse embryonic fibroblasts (MEFs) reconstituted with wild type Ago2 (Ago2 WT) or slicer-defective mutant Ago2 (Ago2 D669A, or “DA,” containing an aspartate to alanine substitution at residue 669)^22–24^. Expression levels of these MD-miRNAs were increased by reconstitution of Ago2 WT (Supplementary Fig. 2b). Importantly, Ago2 D669A mutant only partially induced expression of key MD-miRNAs including miR-107, miR-103, and miR-130a, while expression of miR-148b required Ago2 but not its slicer activity (Supplementary Fig. 2b). While the loss of Dicer also caused a reduction of MD-miRNAs (Supplementary Fig. 2c), these results support a crucial role of Ago2 in the expression of a subset of MD-miRNAs.

While Dicer recognizes the 5′ phosphate end and 2-nucleotide 3′ overhang structure of precursor miRNA for precise and effective biogenesis of miRNAs^30,31^, recent studies have provided different mechanistic insights into the Ago2-mediated processing of miRNA. One of the proposed characteristics of miRNAs processed by Ago2 is that their precursors have a relatively shorter loop size that likely prevents recognition by Dicer. Moreover, these precursors have no mis-matching at position 10 or 11 between guide and passenger strands^32^. The miRNAs with reduced expression in L-Ago2 KO liver tended to have shorter loop sizes than those induced by L-Ago2 KO liver, and had no mis-matching at positions 10 or 11 (Supplementary Fig. 2d–f). This information suggests that there may be structural similarities among MD-miRNAs that require Ago2 for maturation.

We next asked if any of the 25 significant miRNAs, Ago1, or Ago2 might have disease associations proximal to their orthologous human miRNAs and genes. To this end, we first identified mouse-human orthologous miRNAs using data from miRBase^33^ and miROrtho^34^, identifying a total of 27 human miRNAs (some mouse miRNAs do not have clear orthologs or map to two human miRNAs) (Supplementary Table 2). We then examined a large collection of genetic variants associated with 213 diseases and phenotypes collected from the National Human Genome Research Institute (NHGRI) Catalog of Published Genome-Wide Association Studies (GWAS)^35^ that have been expanded to include additional variants in strong linkage disequilibrium (*r*^*2*^ > 0.8) with the tagged variants^36^. This analysis revealed that several of the orthologous human miRNAs have proximal GWAS signal, often for relevant phenotypes (Supplementary Table 3). For example, a genetic variant (rs6953596) located 245 bases away from the gene encoding miR-148a is strongly associated with body mass index (BMI) in African Americans and thus might act by altering gene regulatory mechanisms controlling the expression of miR-148a. In addition, while there is no detectable GWAS signal near Ago1, there is suggestive GWAS signal (*p* = 10^−6^) located within an intron of Ago2 for “Thiazide-induced adverse metabolic effects in hypertensive patients” in African Americans^37^. These results suggest a possible association of Ago2 and miRNAs whose expression is regulated by Ago2 with human metabolic diseases.

### Inactivation of hepatic Ago2 improves systemic glucose metabolism

**Fig. 2:**
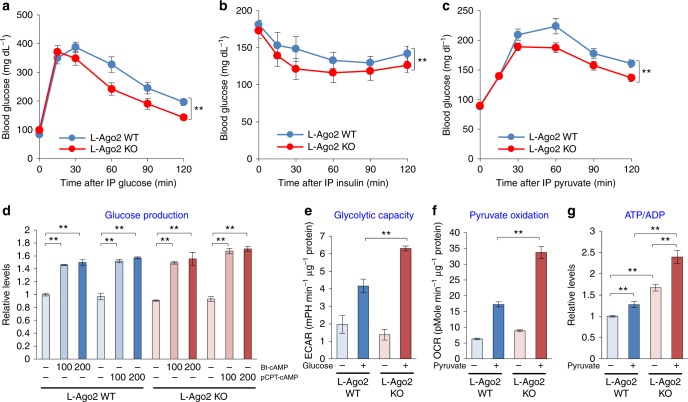
Hepatic Ago2-deficiency improves glucose metabolism. **a** Glucose tolerance test performed in L-Ago2 WT (*n* = 10) and KO (*n* = 8) mice fed NCD at 20 weeks of age. **b** Insulin tolerance test performed in L-Ago2 WT (*n* = 9) and KO (*n* = 6) mice fed NCD at 21 weeks of age. **c** Pyruvate tolerance test performed in L-Ago2 WT (*n* = 9) and KO (*n* = 10) mice fed NCD at 24 weeks of age. **d** Glucose production in primary hepatocytes isolated from L-Ago2 WT and L-Ago2 KO incubated in the absence (*n* = 4 for L-Ago2 WT and L-Ago2 KO, respectively) or presence of 100 or 200 μM Bt-cAMP or pCPT-cAMP (*n* = 2 for L-Ago2 WT and L-Ago2 KO, respectively). **e** Extracellular acidification (ECAR) in the absence or presence of 10 mM glucose in primary hepatocytes isolated from L-Ago2 WT (*n* = 3 for control, *n* = 6 for glucose) and L-Ago2 KO mice (*n* = 3 for control, *n* = 6 for glucose). **f** Mitochondrial oxygen consumption rate (OCR) in the absence or presence of 2 mM pyruvate in primary hepatocytes isolated from L-Ago2 WT (*n* = 3 for control and pyruvate, respectively) and L-Ago2 KO mice (*n* = 3 for control and pyruvate, respectively). **g** The ATP/ADP ratio in primary hepatocytes isolated from L-Ago2 WT and L-Ago2 KO mice in the absence (*n* = 6 for L-Ago2 WT and L-Ago2 KO, respectively) or presence of 5 mM pyruvate (*n* = 6 for L-Ago2 WT and L-Ago2 KO, respectively). Data are shown as the mean ± SEM. *p < 0.05, **p < 0.01

Considering the miRNA enrichment pathway analysis indicated that hepatic Ago2 is implicated in glucose metabolism, we then investigated Ago2’s role in regulating this regard. We observed that L-Ago2 KO mice fed normal chow diet (NCD) exhibited enhanced capacities for glucose metabolism, as assessed by glucose, insulin, and pyruvate tolerance tests (GTT, ITT, and PTT) after 20 weeks of age (Fig. 2a–c). These results suggest that hepatic Ago2 deficiency improves insulin sensitivity and inhibits gluconeogenesis, leading to glucose tolerance. To investigate how Ago2 regulates glucose metabolism, we first examined capacities of hepatic gluconeogenesis in Ago2-deficient primary hepatocytes. Glucose production was similarly induced between genotypes (Fig. 2d). We then asked if Ago2 regulates the fundamental catabolic capacities of hepatocytes. To examine the glycolytic rate, the extracellular acidification rate (ECAR) was determined in primary hepatocytes upon addition of glucose. In the presence of oligomycin, Ago2-deficient hepatocytes showed a higher increase in ECAR compared to controls (Fig. 2e). To determine whether Ago2 regulates oxidation of pyruvate, we measured mitochondrial oxygen consumption rate (OCR) in WT and Ago2-deficient hepatocytes in the presence of pyruvate. Upon the addition of the protonophore carbonyl cyanide 4-(trifluoromethoxy) phenylhydrazone (FCCP), Ago2-deficient hepatocytes greatly upregulated oxygen consumption compared to WT controls (Fig. 2f). In a complementary approach, we also measured ATP content/ADP content in these hepatocytes in the absence or presence of pyruvate. The basal ATP/ADP ratio in Ago2-deficient hepatocytes was higher than that in WT controls, and this difference was even greater upon addition of pyruvate (Fig. 2g). Taken together, these data indicate that hepatic Ago2 functions to enhance gluconeogenesis and suppress glucose oxidation while its inactivation results in increased glucose-driven energy production.

### Hepatic Ago2 regulates glucose metabolism in insulin insufficiency

**Fig. 3:**
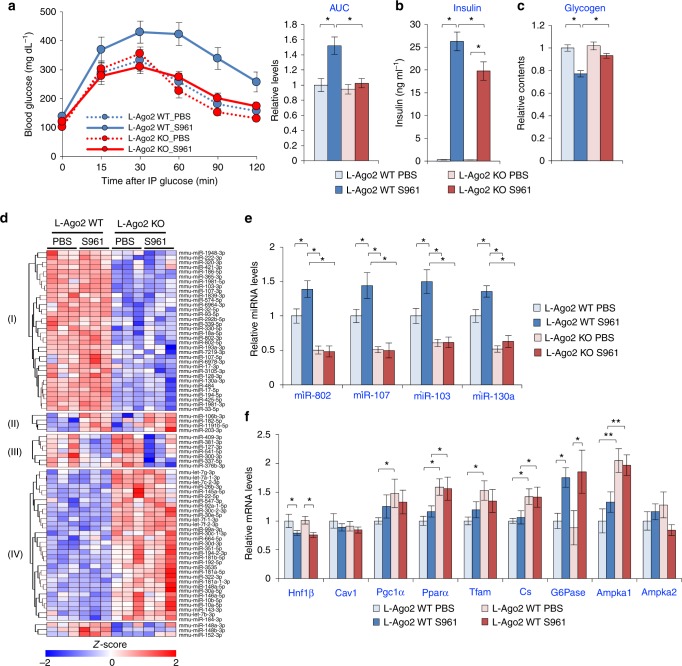
Hepatic Ago2-deficiency prevents S961-induced acute glucose intolerance. **a** Glucose tolerance tests at one-week post treatment of S961 or phosphate-buffered saline (PBS). L-Ago2 WT (*n* = 10 for PBS and n = 13 for S961) and KO (*n* = 11 for PBS and *n* = 14 for S961) mice fed NCD at 9 weeks of age were continuously treated with S961 (10 nM/week) via osmotic pumps. The graph on the right shows an integrated area under the glucose disposal curves (AUC) for each condition. **b** Serum insulin levels after daytime food withdrawal for 6 h in L-Ago2 WT and KO mice at 2 weeks post S961 (*n* = 7, each genotype) or PBS (*n* = 6, each genotype) treatment. **c** Hepatic glycogen contents in L-Ago2 WT and KO mice at 2 weeks post S961 (*n* = 7, each genotype) or PBS (*n* = 5, each genotype) treatment. **d** A heatmap diagram illustrating the differential expression of mature miRNAs in the liver of L-Ago2 WT and KO mice treated with PBS or S961 for 2 weeks. Significant miRNAs differentially expressed between genotypes were identified using DESeq2 (|fold change| > 1.25x and adjusted *p* < 0.0005). Clusters I and IV are miRNAs differentially expressed between L-Ago2 WT (*n* = 6) and L-Ago2 KO (*n* = 6) groups. Clusters II and III are miRNAs differentially expressed by S961 treatment in WT and KO groups, respectively. The log2 expression values were scaled by z-score. **e**, **f** Effect of S961-treatment on expression of MD-miRNAs (**e**) and genes regulating energy metabolism (**f**) in the liver of L-Ago2 WT and KO mice treated with PBS or S961 (*n* = 6, each group) for 2 weeks. Data are shown as the mean ± SEM. *p < 0.05, **p < 0.01

If hepatic Ago2-deficiency improves systemic glucose metabolism by suppressing gluconeogenesis and accelerating glucose oxidation in the liver, other diabetic conditions may also be improved by hepatic Ago2-deficiency. To examine this possibility, we employed a pharmacological model by administering the insulin antagonist peptide, S961 (43 amino acids in length). S961 binds to the insulin receptor and blocks insulin signaling in vivo to acutely induce hyperglycemia^38^. This model allowed us to further assess the role of hepatic Ago2 in glycemic control without the potential confounding effects of body weight and adiposity. After infusing S961 into L-Ago2 WT and L-Ago2 KO mice fed NCD at 12 weeks of age, we examined the effect of hepatic Ago2-deficiency on S961-induced deterioration of glucose metabolism. At this age, while glucose metabolism assessed by GTT was comparable between the genotypes in a PBS-treated control group, S961 treatment caused hyperglycemia in WT mice 1 week after treatment (Fig. 3a), and hyperinsulinemia and glycogenolysis in WT mice after 2 weeks of treatment (Fig. 3b, c). Remarkably, L-Ago2 KO mice are resistant to S961-induced glucose intolerance after 1 week of treatment (Fig. 3a) and S961 treatment of L-Ago2 KO mice resulted in a lower induction of plasma insulin levels and higher hepatic glycogen contents compared with control mice (Fig. 3b, c). These data indicate that inactivation of hepatic Ago2 improves systemic glucose metabolism in the condition of insulin insufficiency.

Expression profiles that assessed the effect of insulin insufficiency on miRNAs in the liver categorized four different classes of miRNAs; (I) dominantly expressed in L-Ago2 WT, (II) induced by S961 in both L-Ago2 WT and KO, (III) suppressed by S961 in both L-Ago2 WT and KO, and (IV) dominantly expressed in L-Ago2 KO (Fig. 3d). Importantly, S961 treatment did not modulate expression levels of miRNAs categorized in class (IV) in both L-Ago2 WT and KO liver. Conversely, several miRNAs in class (I) that contains MD-miRNAs including miR-802, miR-103/107, and miR-130a were strikingly induced by S961 treatment in the WT liver in an Ago2-dependent manner (Fig. 3d, e). These analyses implicate hepatic Ago2-dependent miRNAs play a role in the disruption of glucose metabolism under the condition of insulin insufficiency. Despite the decrease of miR-802 and miR-103/107 expression in the Ago2-deficient condition, expression levels of their known targets, *Hnf1β* and *Cav1*, were comparable between the genotypes even in PBS-treated groups at this age (Fig. 3f). While expression of glucose-6-phosphatase (*G6Pase*), a gluconeogenic gene, was similarly induced between the genotypes (Fig. 3f), expressions of genes critical for mitochondrial function, such as *Pgc1α*, peroxisome proliferator-activated receptor alpha (*Pparα)*, mitochondrial transcription factor (*Tfam*), and citrate synthase (*Cs*) were higher in the liver of L-Ago2 KO mice. These results, along with the Ago2-deficient hepatocyte analyses, suggest that Ago2 regulates the program of a subset of miRNAs involved in energy metabolism, which may be associated with enhancement of gluconeogenesis and suppression of glycolysis and hepatic mitochondrial oxidation induced by insulin insufficiency.

### Critical roles of hepatic Ago2 in energy metabolism on high-fat-diet challenge

**Fig. 4:**
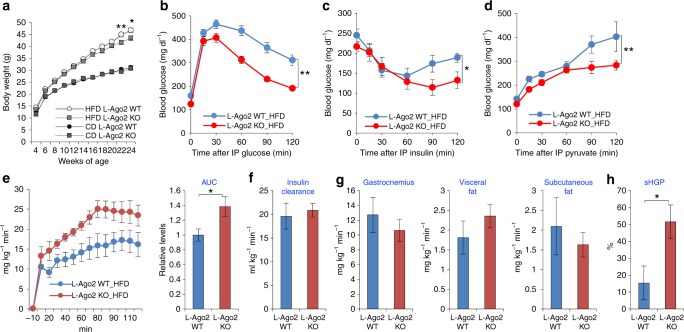
Ago2-deficiency in the liver improves glucose metabolism in obesity. **a** Body weights of L-Ago2 WT (*n* = 16) and KO (*n* = 17) mice fed HFD, and L-Ago2 WT (*n* = 15) and KO (*n* = 11) mice fed CD, starting in 4 weeks of age. **b** GTT performed in L-Ago2 WT (*n* = 16) and KO (*n* = 17) mice fed HFD at 20 weeks of age. **c** ITT performed in L-Ago2 WT (*n* = 16) and KO (*n* = 17) mice fed HFD at 14 weeks of age. **d** PTT performed in L-Ago2 WT (*n* = 8) and KO (*n* = 8) mice fed HFD at 17 weeks of age. **e**–**h** Hyperinsulinemic-euglycemic clamp studies performed in L-Ago2 WT (*n* = 6) and L-Ago2 KO (*n* = 10) mice fed HFD for 20 weeks. **e** Glucose infusion rates (GIR) throughout the clamp procedures. The graph on the right shows an integrated area under curves (AUC) of GIR. **f** Insulin clearance levels during the clamp. **g** Tissue glucose uptakes in gastrocnemius muscle, visceral fat, and subcutaneous fat tissues. **h** Suppression of hepatic glucose production (sHGP) during the clamp. Data are shown as the mean ± SEM. *p < 0.05, **p < 0.01

We next asked if nutrient challenge might accentuate Ago2’s role in metabolic regulation. We thus employed a high-fat diet (HFD)-induced obesity model that induces insulin resistance, glucose intolerance, and hepatic steatosis. We placed L-Ago2 KO, and L-Ago1 KO, and control WT mice on HFD or a control diet (CD), commencing at 4 weeks of age (Fig. 4a and Supplementary Fig. 3a). Of note, the body weights of the HFD-fed L-Ago2 KO mice became lower than that of controls and the difference reached statistical significance at 22 weeks of age (Fig. 4a). Conversely, the body weight of L-Ago1 KO mice was comparable to controls in the HFD condition (Supplementary Fig. 3a). The improvements in glucose metabolism observed in the HFD-fed L-Ago2 KO were even more pronounced when compared to the CD-feeding condition, as assessed by GTT, ITT, and PTT (Fig. 4b–d, Supplementary Fig. 4a and b). These improvements were not observed in L-Ago1 KO fed HFD (Supplementary Fig. 3b–f). Consistent with improved systemic glucose tolerance and insulin sensitivity in L-Ago2 KO mice, HFD-induced pancreatic β-cell proliferation and islet hypertrophy were attenuated compared to L-Ago2 WT mice (Supplementary Fig. 4c–g), supporting that hepatic Ago2-deficiency improves insulin sensitivity in the pathogenesis of obesity.

To further demonstrate the role of hepatic Ago2, we performed the hyperinsulinemic-euglycemic clamp study to examine the whole-body glucose metabolism and insulin sensitivity. Glucose infusion rates (GIR) during the clamp studies indicated that L-Ago2 KO mice required significantly higher levels of glucose infusion to maintain blood glucose consistent with increased insulin sensitivity (Fig. 4e and Supplementary Fig. 4h). Insulin clearance and glucose uptakes in gastrocnemius muscle, visceral and subcutaneous fat, brown adipose tissue, and heart were comparable (Fig. 4f, g and Supplementary Fig. 4j). Conversely, hepatic glucose production was significantly suppressed in L-Ago2 KO mice (Fig. 4h and Supplementary Fig. 4k). These studies indicate that the liver is the main locus responsible for improving systemic insulin sensitivity and glucose metabolism in L-Ago2 KO mice.

**Fig. 5:**
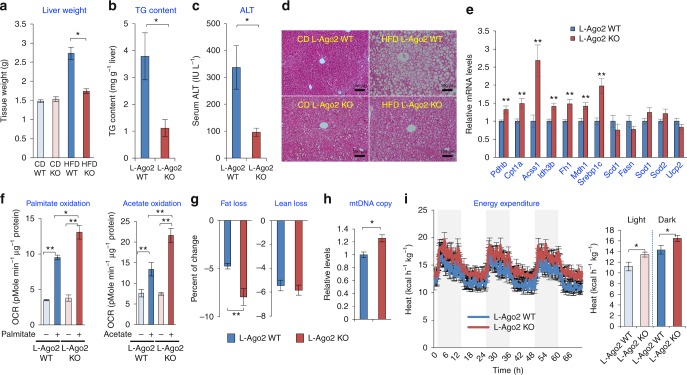
Ago2-deficiency in the liver prevents hepatic steatosis with enhanced energy expenditure. **a** Liver weight in L-Ago2 WT mice fed CD (*n* = 5), L-Ago2 KO mice fed CD (*n* = 4), L-Ago2 WT mice fed HFD (*n* = 5), and L-Ago2 KO mice fed HFD (*n* = 5) at 30 weeks of age. **b**, **c** Liver triglyceride (TG) contents (**b**) and serum ALT levels (**c**) in L-Ago2 WT (*n* = 7) and L-Ago2 KO (*n* = 5) mice fed HFD at 23 weeks of age. **d** H&E-stained sections of the liver in each genotype at 30 weeks of age. Scale bar, 100 μm. **e** Expression levels of key mRNAs involved in energy metabolism in the liver of L-Ago2 WT (*n* = 8) and L-Ago2 KO (*n* = 5) mice fed HFD at 23 weeks of age. **f** Levels of β-oxidation in the presence of 0.12 mM palmitate and mitochondrial OCR in the presence of 5 mM acetate in primary hepatocytes isolated from L-Ago2 WT (*n* = 3 for control, *n* = 3 palmitate and acetate, respectively) and L-Ago2 KO mice (*n* = 3 for control, *n* = 3 palmitate and acetate, respectively). **g** Effects of a 14-h fast on fat mass, and lean body mass in L-Ago2 WT (*n* = 9) and KO (*n* = 10) mice fed HFD at 20 weeks of age. **h** Copy numbers of mtDNA in L-Ago2 WT (*n* = 7) and KO (*n* = 5) mice fed HFD at 23 weeks of age. **i** Energy expenditure in L-Ago2 WT (*n* = 8) and KO (*n* = 8) mice fed HFD at 16 weeks of age. Data are shown as the mean ± SEM. *p < 0.05, **p < 0.01

Importantly, the liver of L-Ago2 KO mice was characterized by lowered liver weights and triglyceride content, accompanied by lower serum ALT levels on HFD (Fig. 5a–c). In addition, plasma triglyceride levels were lower in L-Ago2 KO mice fed HFD, while those of cholesterol, phospholipids, and free fatty acids were comparable between the genotypes (Supplementary Fig. 5a–d). There was also a reduction in hepatic fatty infiltration as visualized by haematoxylin and eosin (H&E) staining (Fig. 5d). While levels of genes involved in lipid biosynthesis such as stearoyl-CoA desaturase-1 (*Scd1*) and fatty acid synthease (*Fasn*) were comparable between the genotypes, those of carnitine palmitoyltransferase 1A (*Cpt1a*) and acetyl-coenzyme A synthetase 2-like (*Acss1*) that mediate catabolic processes of fatty acids were increased in the livers of L-Ago2 KO mice (Fig. 5e). Consistently, mitochondrial OCR in response to palmitate and acetate whose circulating levels are positively correlated with obesity and its related sequelae^39^ was significantly higher in Ago2-deficient hepatocytes compared to controls (Fig. 5f). These cellular phenotypes could promote reduction of hepatic triglyceride accumulation and lowered hepatic steatosis levels we have observed in the liver of L-Ago2 KO mice fed HFD. Quantitative Magnetic Resonance technology (EchoMRI) analyses revealed that total body fat mass in L-Ago2 KO mice is lower than in WT controls (44.3% fat reduction in L-Ago2 KO: *p* < 0.05, *t*-test) (Supplementary Fig. 5e), while lean mass composition of L-Ago2 KO mice was slightly higher than that of control mice on HFD at 20 weeks of age (Supplementary Fig. 5f). In addition, the ability to utilize fat mass, which was calculated by measurements of fat and lean compositions before and after an overnight fast, was higher in L-Ago2 KO mice on HFD compared to WT controls (1.675-fold higher in L-Ago2 KO: *p* < 0.01, *t*-test) (Fig. 5g). In support of these observations, there was an increased copy number of mitochondrial-DNA (mtDNA) in the Ago2-deficient liver in obesity (Fig. 5h). Consistently, rates of energy expenditure of L-Ago2 KO mice fed HFD for 12 weeks were significantly higher than those of controls (Fig. 5i and Supplementary Fig. 5g and h), despite no significant changes in body weight, total physical activity, food intake, or amounts of fecal lipids between genotypes (Supplementary Fig. 5i–l). We performed similar experiments with L-Ago1 WT and L-Ago1 KO mice fed HFD and found that there were no differences in the regulation of energy homeostasis between the genotypes (Supplementary Fig. 3g–m). Taken together, these data indicate that inactivation of Ago2, but not Ago1, in the liver increases mitochondrial capacity and energy expenditure, which appears to link to improvement of obesity-associated pathophysiology.

### Ago2-mediated RNA silencing regulates expression of genes involved in energy metabolism

**Fig. 6:**
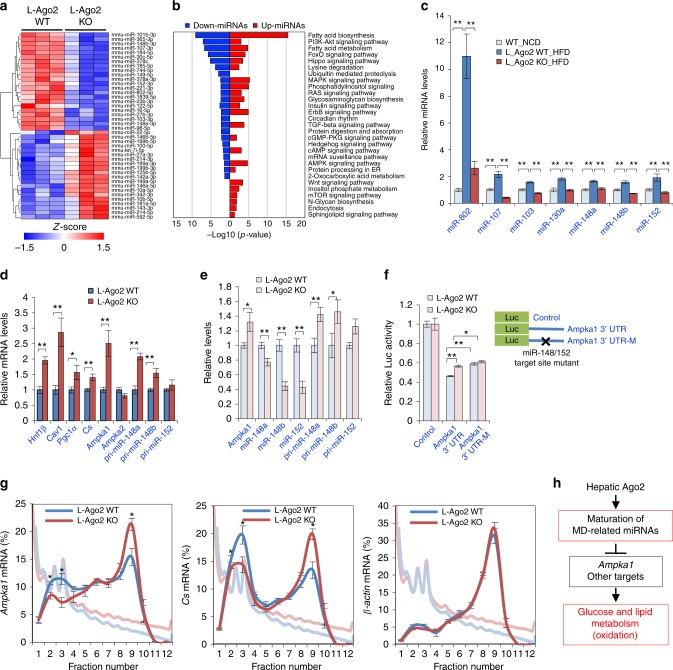
Hepatic Ago2 regulates expression of MD-miRNAs and *Ampka1* in obesity. **a** A heatmap diagram illustrating the differential expression of mature miRNAs in the liver of L-Ago2 WT (*n* = 3) and L-Ago2 KO (*n* = 3) mice fed HFD for 16 weeks. Significant miRNAs differentially expressed between L-Ago2 WT and L-Ago2 KO groups were identified using DESeq2 (|fold change| > 2x and adjusted *p* < 0.05) and plotted as a heatmap using z-score. **b** Metabolic pathway enrichment analysis of miRNAs significantly down-regulated (blue) and up-regulated (red) miRNAs in the liver of L-Ago2 KO mice fed HFD for 16 weeks. These miRNAs were queried to calculate the most enriched KEGG pathways using DIANA-mirPath web-server (*p* < 0.05 and MicroT threshold < 0.8). Pathways unrelated to liver functions were excluded in this pyramid plot. **c** Expression levels of specific MD-miRNAs in the liver of L-Ago2 WT mice (*n* = 8) fed NCD at 25 weeks of age, and L-Ago2 WT (*n* = 7) and L-Ago2 KO (*n* = 5) mice fed HFD at 23 weeks of age. **d** Expression levels of MD-miRNAs’ target mRNAs and pri-miRNAs in the liver of L-Ago2 WT (*n* = 7) and L-Ago2 KO (*n* = 5) mice fed HFD at 25 weeks of age. **e** Compared expression levels of *Ampka1*, miR-148/152, and their pri-miRNAs in primary hepatocytes isolated from L-Ago2 WT and L-Ago2 KO mice. **f** Relative luciferase activity by which *Ampka1* 3'﻿ UTR with or without harboring a mutation at miR148/152 putative target site was assessed in primary hepatocytes isolated from L-Ago2 WT (*n* = 4 for control, *n* = 6 for *Ampka1* 3′ UTR and *Ampka1* 3′ UTR-M, respectively) and L-Ago2 KO mice (*n* = 4 for control, *n* = 6 for *Ampka1* 3′ UTR and *Ampka1* 3′ UTR-M, respectively). **g** Quantification of *Ampka1*, *Cs*, and *β-actin* mRNA levels in fractions collected from polysome profiles of primary hepatocytes isolated from L-Ago2 WT and L-Ago2 KO mice. The graphs show the quantification of the results. **h** A proposed role of hepatic Ago2 in the regulation of glucose and lipid metabolism in the liver. Data are shown as the mean ± SEM. *p < 0.05, **p < 0.01

To investigate molecular mechanisms by which Ago2 orchestrates hepatic energy metabolism, we additionally profiled hepatic miRNA expression under the condition of HFD (Fig. 6a). Utilizing the list of significant miRNAs on HFD, the miRNA target pathway enrichment analysis revealed that hepatic Ago2 deficiency affected several functional clades such as glucose and lipid metabolism and protein translation regulation (Fig. 6b). While Ago2 protein levels were slightly decreased in the liver of mice fed HFD (Supplemental Fig. 6a), several MD-miRNAs were highly induced in the liver of L-Ago2 WT mice fed HFD and a leptin-deficient (*ob/ob*) obese mice (Fig. 6c and Supplemental Fig. 6b). Importantly, these miRNAs are constantly decreased in that of L-Ago2 KO mice (Fig. 6a, c). Consistent with these observations, expression levels of their known target mRNAs, such as *Hnf1β*, *Cav1*, and *Pgc1α*, are increased in L-Ago2 KO liver (Fig. 6d). Of note, expression levels of these genes were comparable between L-Ago1 WT and KO liver (Supplementary Fig. 3n).

To explore targets of Ago2-dependent MD-miRNAs for metabolic regulation, we then took a bioinformatics approach with miRNA sequencing data obtained under lean, HFD, and S961-treated conditions (Supplementary Fig. 6c and d). With the Ago2-dependent miRNAs, we extracted lists of predicted conserved target genes involved in energy metabolism from the widely used TargetScan 7.1 website. This analysis identified a subset of genes, including *Ampka1* (also known as *Prkaa1*, a catalytic subunit of AMPK that plays a critical role in AMPK activation) and *Cs*, that have 3′ untranslated region (UTR) containing multiple target sites for Ago2-dependent miRNAs including miR-148a/152 known to evoke hyperlipidemia, hypercholesteremia, and atherosclerosis^14,15^ (Supplementary Fig. 6e and f). As AMPK is known as a critical regulator of energy metabolism, we further assessed the role of Ago2 in *Ampka1* expression. Analyzing a public database of photoactivatable ribonucleoside-enhanced crosslinking and immunoprecipitation (PAR-CLIP) with Ago2^40^ revealed that Ago2 binds to the region of the *Ampka1* 3′ UTR that contains binding sites of Ago2-dependent miRNAs including miR-148/152 and miR-130a (Supplementary Fig. 6g). Consistent with these findings, we confirmed the induction of *Ampka1* expression, accompanied by the reduction of miR-148a, miR-148b, and miR-152, in the liver of L-Ago2 KO mice fed HFD and in Ago2-deficient primary hepatocytes (Fig. 6d, f). We next conducted luciferase assays in which *Ampka1’s* 3′ UTR, with or without harboring a mutation at the miR-148/152 target site, was sub-cloned into luciferase expression vector. The luciferase activity of each was measured in primary hepatocytes isolated from L-Ago2 WT and KO mice. Luciferase activity was higher in Ago2 KO hepatocyte transfected with Ampkα1 3′ UTR without a mutation compared with Ago2 WT hepatocyte, but the induction in Ago2 KO hepatocyte disappeared in the setting with mutated  *Ampka1* 3' UTR (Fig. 6f). These analyses demonstrated that miR-148/152 are involved in suppression of *Ampka1* expression in a manner dependent on Ago2 and a miR-148/152 target site (Fig. 6f). As miRNA inhibits the translation of target mRNAs through RNA silencing, we additionally asked if Ago2-deficiency affects translation of genes having target sites of MD-miRNAs by investigating polysome-bound mRNA expression patterns. Expression levels of polysome-bound *Ampka1* and *Cs* were enriched in Ago2-deficient primary hepatocytes, while those of *β-Actin* were comparable (Fig. 6g). Taken together, these findings further confirm that hepatic Ago2-mediated MD-miRNA expression and RNA silencing are linked to expressional regulation of genes involved in energy metabolism (Fig. 6h).

### Hepatic Ago2 regulates energy consumption associated with AMPK activation

**Fig. 7:**
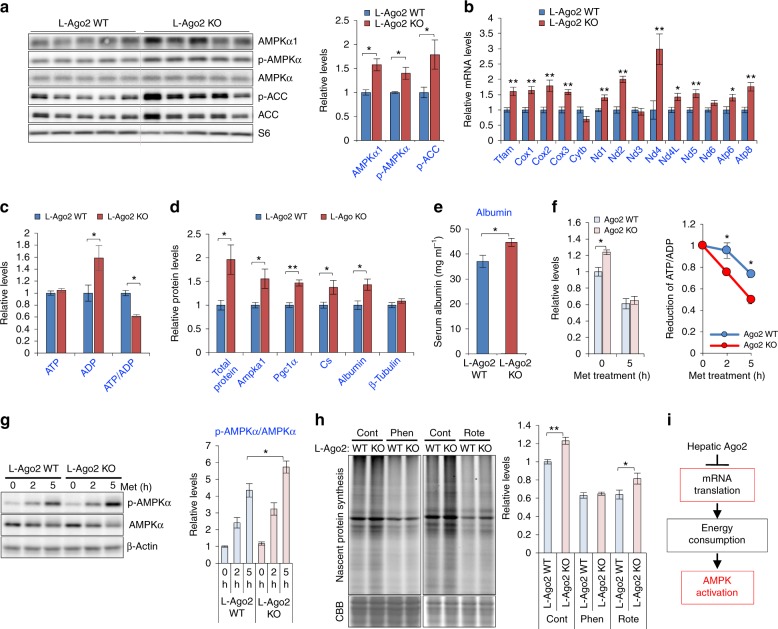
Hepatic Ago2-deficiency enhances energy expenditure associated with protein synthesis and AMPK activation. **a**, **b** Western blot analyses of AMPK expression and activation (**a**) and mRNA expression of the Tfam-mitochondrial genes (**b**) in the liver of L-Ago2 WT (*n* = 5) and L-Ago2 KO (*n* = 5) fed HFD at 25 weeks of age. **c** ATP, ADP, and ATP/ADP ratio levels in L-Ago2 WT (*n* = 5) and L-Ago2 KO (*n* = 5) mice fed HFD at 25 weeks of age. ATP/ADP ratio levels were independently measured with a distinct procedure from the ATP and ADP assays. **d** Western blot analysis of total and specific protein levels normalized by 12S-genomic DNA in the liver of L-Ago2 WT (*n* = 5) and KO (*n* = 5) mice fed HFD at 30 weeks of age. **e** Serum albumin levels in L-Ago2 WT (*n* = 8) and L-Ago2 KO (*n* = 8) mice fed HFD at 25 weeks of age. **f** Energy consumption rate measured in primary hepatocytes isolated from L-Ago2 WT (*n* = 4, *n* = 4, *n* = 3 for 0, 2 h, 5 h, respectively) and L-Ago2 KO (*n* = 4, *n* = 4, *n* = 4 for 0, 2 h, 5 h, respectively) mice in the presence of 1 mM metformin. **g** Effect of Ago2-deficiency on expression of AMPK activation in primary hepatocytes isolated from L-Ago2 WT (*n* = 8, *n* = 8, *n* = 8 for 0, 2 h, 5 h, respectively) and L-Ago2 KO (*n* = 8, *n* = 8, *n* = 8 for 0, 2, 5 h, respectively) mice in the presence of 1 mM metformin. The graphs show the quantification of the results. **h** Effect of Ago2 on nascent protein synthesis. Primary hepatocytes isolated from L-Ago2 WT and L-Ago2 KO mice were treated with or without 200 μM Phenformin (Phen) or 10 μM Rotenone (Rote) for 5 h (*n* = 15 for control, *n* = 9 for Phen, *n* = 6 for Rote). **i** A proposed role of hepatic Ago2 in suppression of protein translation, leading to AMPK activation. The graphs show the quantification of the results. Data are shown as the mean ± SEM. *p < 0.05, **p < 0.01

We next examined AMPKα1 protein levels and noticed that Ago2 deficiency increased not only the protein levels but also activity of AMPK, assessed by phosphorylation levels of AMPKα, AMPKβ, and an AMPK substrate, Acetyl-CoA carboxylase (ACC), in the liver of L-Ago2 KO mice fed HFD and treated with S961 (Fig. 7a and Supplementary Fig. 7a and b). In agreement with the activation of AMPK, other AMPK substrates, UNC-51-like kinase 1 (ULK1), and Mitochondrial fission factor (MFF) are increased in the liver of L-Ago2 KO mice (Supplementary Fig. 7a), suggesting enhanced autophagy/mitophagy and improved mitochondrial quality in the Ago2-deficient liver in obesity. Consistently, expression levels of the Tfam-mitochondrial gene pathway are increased in the liver of L-Ago2 KO mice fed HFD (Fig. 7b).

AMPK is activated when cellular energy level becomes low. Indeed, we found a profound induction of ADP levels in the Ago2-deficient liver, while ATP levels are comparable between the genotypes, leading to a reduction of ATP/ADP ratio in the liver of L-Ago2 KO mice fed HFD (Fig. 7c). While Ago2-deficiency enhances capacity for mitochondrial oxidation and ATP production in hepatocytes (Figs. 2g, 5f), systemic energy expenditure is also increased (Fig. 5i). Therefore, we hypothesized that both energy production and consumption are enhanced in the liver of L-Ago2 KO mice compared to their controls. Since a main function of Ago2 is to suppress protein translation, which is one of the most energy consuming cellular processes, through RNA silencing, we investigated the effect of Ago2-deficiency on protein synthesis in the liver. Levels of total and specific proteins normalized by DNA contents were higher in the liver of L-Ago2 KO mice (Fig. 7d and Supplementary Fig. 7c). Similarly, examination of the levels of hepatic and serum albumin, one of the most abundant circulating proteins produced by the liver, revealed that the albumin levels were increased in L-Ago2 KO mice compared to their controls (Fig. 7d, e). These observations suggest that enhanced protein synthesis in the liver may result in a lowered ATP/ADP ratio and AMPK activation in L-Ago2 KO mice.

To further examine if Ago2 deficiency accelerates cellular energy consumption associated with protein synthesis, we treated primary hepatocytes with metformin, which is an anti-diabetic drug and inhibits mitochondrial respiratory-chain complex I activity restricting ATP generation^41^, and measured ATP/ADP levels. Consistent with enhanced capacity for energy production in Ago2-deficiency (Fig. 2g), relative ATP/ADP levels were higher in Ago2-deficient hepatocytes compared to controls, however, the levels were rapidly decreased post-metformin treatment, suggesting a higher energy consumption rate in Ago2-deficiency (Fig. 7f). Consistently, levels of metformin-induced AMPK activation in Ago2-deficient hepatocytes were significantly higher than that in controls (Fig. 7g). To directly investigate the effect of Ago2-deficiency on protein synthesis in hepatocytes, we measured the levels of nascent protein synthesis. Compared to WT controls, the levels were significantly increased in Ago2-deficient hepatocytes (Fig. 7h). By restricting energy supply using phenformin and rotenone, both of which inhibit mitochondrial respiratory-chain complex I activity, the levels of nascent protein synthesis in Ago2-deficient hepatocytes were equivalent or still higher compared to those in controls (Fig. 7g).

Since Ago2's  slicer activity uniquely regulates RNA silencing, we then asked if the slicer activity is involved in the regulation of energy consumption and AMPK activation. Ago2-deficient MEFs were characterized by enhanced expression of Ampka1, and reconstitution of the MEFs with WT Ago2 suppressed expression of both *Ampka1* mRNA and AMPKα protein, whereas the Ago2 D669A mutant did not (Supplementary Fig. 8a). We also observed that AMPK activity, assessed by phosphorylation levels of AMPKα and ACC, was higher in Ago2-deficient MEFs under serum starvation condition (Supplementary Fig. 8a). While activated AMPKα is known to suppress mRNA translation^42^, levels of nascent protein synthesis in Ago2-deficient MEFs are increased compared to the cells reconstituted with WT Ago2 (Supplementary Fig. 8b). To investigate if enhanced protein synthesis reasons AMPK activation in Ago2 deficiency, we treated MEFs with cycloheximide (CHX), a protein synthesis inhibitor, and monitored AMPK activation. Inhibition of protein synthesis resulted in reduction of AMPK activation in WT MEFs, and the effect became more robust in Ago2-deficient MEFs, indicating that Ago2 suppresses protein synthesis-mediated energy consumption (Supplementary Fig. 8c). Taken together, these results indicate that, in addition to an increase of *Ampka1* expression, there is enhanced energy consumption associated with protein synthesis, leading to the lowered ATP/ADP ratio, which appears to enhance AMPK activation in Ago2-deficient conditions (Fig. 7i).

### Hepatic Ago2 deficiency improves glucose metabolism in a hepatic *Ampka1*-deficient condition

**Fig. 8:**
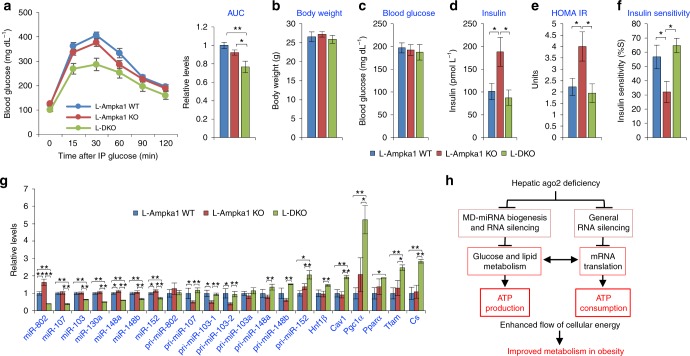
Effects of hepatic Ago2-deficiency on glucose metabolism in liver-specific *Ampka1*-deficient mice. **a** GTT performed in L-Ampka1 WT (*n* = 17), L-Ampka1 KO (*n* = 14), and L-DKO (*n* = 6) mice fed HFD for 5 weeks. **b**–**f** Body weight (**b**), levels of blood glucose (**c**) and plasma insulin (**d**), and calculated HOMA IR (**e**) and insulin sensitivity (**f**) in L-Ampka1 WT (*n* = 10), L-Ampka1 KO (*n* = 8), and L-DKO (*n* = 6) mice fed HFD for 6 weeks. **g** Expression levels of miRNAs, pri-miRNAs, and miRNAs in the liver of L-Ampka1 WT (*n* = 8), L-Ampka1 KO (*n* = 8), and L-DKO (*n* = 3) mice fed HFD for 8 weeks. Data are shown as the mean ± SEM. *p < 0.05, **p < 0.01. **h** A proposed molecular mechanism by which hepatic Ago2-deficiency improves energy metabolism in the pathogenesis of obesity. Ago2-mediated miRNA expression and RNA silencing coordinately suppress mitochondrial oxidation and protein translation, which result in lowered energy supply and consumption. Conversely, hepatic Ago2-deficiency enhances generation of energy from glucose and lipid for protein synthesis, leading to higher ADP and AMP amounts. As a result, the AMPK pathway is activated, leading to improvement of mitochondrial functions and metabolism

While hepatic Ago2-deficiency reduces expression of a specific repertoire of MD-miRNAs of which some of them target *Ampka1*, it is obvious that changes in expression of these miRNAs also affects translation of other target mRNAs. Similarly, enhanced protein synthesis in the liver of L-Ago2 KO mice must influence not only AMPK activation but also other cellular events linked metabolic regulation. To clarify the role of *Ampka1* in the metabolic alterations in L-Ago2 KO mice, we generated liver-specific Ampka1- and Ago2-deficient mice (L-DKO mice) and placed them and their control groups, L-Ampka1 WT and L-Ampka1 KO mice, on HFD for analyses of glucose metabolism. While no significant difference was observed in body weight and fasting blood glucose levels among these three groups, L-DKO mice exhibited enhanced glucose tolerance in the condition of HFD feeding for 5 weeks (Fig. 8a–c). Plasma insulin levels of L-Ampka1 KO mice were higher than those in L-Ampka1 WT mice and the levels were drastically decreased in L-DKO mice on HFD (Fig. 8d). These results indicate that inactivation of hepatic Ago2 can improve glucose metabolism even in an *Ampka1*-deficient condition where insulin resistance occurs (Fig. 8d–f). Consistently, expression levels of a specific repertoire of MD-miRNAs were constantly decreased in the liver of L-DKO mice, while levels of their targets, *Hnf1β*, *Cav1*, and *Pgc1α,* and genes critical for enhancing mitochondrial function were higher in the liver of L-DKO mice compared to L-Ampka1 WT or L-Ampka1 KO mice (Fig. 8g). Taken together, these results suggest that the effect of hepatic Ago2-deficiency on glucose metabolism overrides that of AMPKα1 functions and that a cellular condition which leads to activation of AMPK, but not a direct activation per se, is an important factor that improves glucose metabolism observed in L-Ago2 KO mice.

## Discussion

The role of RNA silencing in suppressing mRNA translation gives rise to the intriguing hypothesis that the RNA silencing machinery might be tightly integrated with the regulation of basal metabolic activity and energy homeostasis, as mRNA translation requires a massive amount of energy. In this study, we uncovered that hepatic Ago2 regulates expression of specific miRNAs that silence genes critical for glucose and lipid metabolism and reduces mRNA translation. These Ago2’s functions appear to intimately be linked to energy metabolism by balancing energy production and consumption through regulating specific miRNA expression and general mRNA translation. Changes in hepatic Ago2-mediated energy balance between energy production and consumption in response to nutrient challenges appears to contribute to the pathogenesis of obesity-associated sequelae (Fig. 8h).

Although Ago1 and Ago2 share functional similarities in RNA silencing, our study provides evidence that Ago2 has a distinct role in metabolic regulation. Hepatic Ago1 is dispensable for obesity-induced pathophysiology, as deletion of hepatic Ago1 did not affect diet-induced weight gain, glucose tolerance, or insulin sensitivity. This, in turn, highlights one of the unique functions of Ago2 in regulating the specific miRNA expression and mRNA silencing. While Ago2-deficiency in the liver affects expression of a small proportion of miRNAs, we demonstrated that Ago2 play a critical role in expression of a subset of specific miRNAs, including miR-802, miR-103/107, and miR-148a/152, which are known to negatively impact glucose and lipid metabolism. In addition, expression of these miRNAs is enhanced in response to the energy stress conditions of lower insulin availability or sensitivity, in an Ago2-dependent manner. Under these stress conditions, hepatocytes are normally programmed to stimulate glucose production, triglyceride synthesis, and the assembly and secretion of very low-density lipoprotein particles. Hepatic Ago2 is likely integrated into this program through the generation of selective miRNAs, and by mediating subsequent RNA silencing. While this mechanism may be beneficial for the maintenance of systemic energy homeostasis during hypoglycemia, hypermotility, starvation, and developmental processes, it could also accelerate the development of metabolic diseases in excess nutrient conditions. When each of the four Ago proteins are ablated constitutively in mice, only the loss of Ago2 causes embryonic lethality, whereas loss of other three Ago proteins is dispensable for animal development^24,43–46^. Importantly, Ago2’s slicer activity is required for embryonic and perinatal development^21^. It is reasonable to consider a model where Ago2’s unique function regulates energy metabolism not only in the liver but also in other organs during development and in adulthood. This may, at least in part, explain the universal importance of Ago2 in such a diverse array of mammalian organs.

In this study, we demonstrated that Ago2-mediated RNA silencing connects the regulation of energy supply with protein biosynthesis. This mechanism may be the core of a vicious cycle in disrupted energy metabolism in the obese liver. In this setting, despite a robust activation of the mTORC1 pathway, protein biosynthesis is progressively suppressed, which is a paradox of mRNA translation^6,47^. While obesity is traditionally considered a state of over-nutrition, recent studies suggest that the obese liver may, in some aspects, resemble a condition of energy deprivation in which proper catabolic processes are impaired due to the repression of oxidative phosphorylation pathways and mitochondrial gene expression^7,48^. Consistently, obesity is also known to induce defects in autophagy in the liver, which leads to poor mitochondrial quality control^49–51^. As a result, protein biosynthesis may be impaired due to under-powered energy supply even during the activation of the mTORC1 pathway, leading to further accumulation of energy sources. Conversely, hepatic Ago2-deficiency increases expression of key metabolic genes including *Ampka1* with enhanced cellular energy consumption that can lead to lower ATP/ADP ratio. This condition can amplify activation of AMPK and its substrates ULK1, MFF, and Pgc1α, leading to improved mitochondrial capacity and quality, which in turn generates sufficient energy for protein biosynthesis. Of note, Ago2 is also known to regulate mRNA silencing through interacting with exonuclease complexes, the Ccr4-Not and Pan2-Pan3 complexes^52^, in a miRNA-independent manner, which likely contributes to suppression of protein translation and energy metabolism in the liver. These Ago2-mediated molecular events may solve the paradox of protein biosynthesis in the obese liver, demonstrate a new mechanism in the regulation of basal metabolic activity, and provide a novel therapeutic target for metabolic diseases.

In conclusion, our results highlight that Ago2 uniquely regulates energy production and consumption in the liver, and suggest hepatic Ago2-mediated RNA silencing is a key regulator of glucose and lipid metabolism during the pathogenesis of obesity. We also posit that there may be important translational implications for our findings, especially in the design of therapeutic interventions, to target modulation of a spectrum of Ago2-dependent miRNA-mediated events, in chronic metabolic disorders, such as diabetes, fatty liver diseases, and other obesity-associated sequelae.

## Methods

### Mice

Animal care and experimental procedures were performed according to procedures approved by the animal care committees of Cincinnati Children’s Hospital Medical Center. *Ago1*^*fl/fl*^, *Ago2*^*fl/fl*^, and *Albumin*^*cre/cre*^ were obtained from the Jackson Laboratory (Stock No: 019001, 016520, and 003574, respectively). *Ampka1*^*fl/f*^ mice were kindly provided by Dr. Basilia Zingarelli. All mice used in this study were on C57BL/6 background. Mice were placed on a high-fat diet (HFD: 60% fat, 20% protein, and 20% carbohydrate kcal; Research Diets #D12492) for a diet induced obesity model, a control diet (CD: 10% fat, 20% protein, and 70% carbohydrate; Research Diets #D12450), or normal chow diet (NCD: 29% Protein, 13% Fat and 58% Carbohydrate kcal; LAB Diet #5010) beginning at 4 weeks of age ad libitum with free access to water. For acute insulin-resistant model, S961, an insulin receptor antagonist, was kindly provided by Dr. Lauge Schaffer^38^. The ALZET osmotic pump were used to deliver 10 nM S961 or vehicle (PBS) in a 2 weeks period. GTTs were performed by intraperitoneal glucose injection (1.5 g kg^−1^) following an overnight food withdrawal for 14 h. ITTs were performed by intraperitoneal insulin injection (0.75 IU kg^−1^ for lean mice, 1 IU kg^−1^ for obese mice) following a daytime food withdrawal for 6 h. PTTs were performed by intraperitoneal sodium pyruvate injection (Sigma-Aldrich, 2 g kg^−1^) following an overnight food withdrawal for 16 h. Body composition was analyzed by EchoMRI™-100H instrument (Echo Medical Systems)^53^. To measure body composition after fasting, food was removed from mice for 16 h. To analyze fecal lipid excretion, lipid content of feces was extracted using chloroform:methanol (2:1) and air-dried under a fume hood. Mouse serum albumin levels were measured using an ELISA kit (Abcam). Mouse plasma insulin levels were measured using Mouse Ultrasensitive Insulin ELISA kit (ALPCO). Lipid profiling was also performed by University of Cincinnati’s MMPC Core. Energy expenditure was measured by using PhenoMaster (TSE Systems)^54^. Hyperinsulinemic-euglycemic clamp studies were performed at University of Michigan Animal Phenotyping Core.

### Biochemical reagents and antibodies

All biochemical reagents were purchased from Sigma-Aldrich unless otherwise indicated. Antibodies against, JNK1 (SC-1648, 1:2500), Dicer (SC-30226, 1:2500), Akt (SC-8312, 1:2500), phospho-Akt (Ser473) (SC-7985-R, 1:2500), PGC-1α (SC-13067, 1:2500), β-actin (SC-130656, 1:5000), and β-tubulin (SC-9104, 1:5000) were from Santa Cruz Biotechnology. Anti-Acc (3662, 1:2500), anti-phospho-Acc (Ser79) (11818, 1:2500), Anti-AMPKα (5832, 1:2500), Anti-phospho-AMPKα (Thr172) (2535, 1:2500), anti-AMPKβ (4250, 1:2500), anti-phospho-AMPKβ (Ser108) (4181, 1:2500), anti-phospho-ULK1 (Ser555) (5869, 1:2500), anti-phospho-ULK1 (Ser317) (12753, 1:2500), anti-ULK1 (8054, 1:2500), anti-phospho-MFF (Ser146) (49281, 1:2500), anti-MFF (86668, 1:2500), anti-Ago2 (2897, 1:2500), anti-Ago1 (5053, 1:2500), anti-S6 Ribosomal Protein (2217, 1:2500) and anti-phospho-JNK (Thr183/Tyr185) (1:2500), anti-Albumin (4929, 1:2500), anti-Citrate Synthase (14309, 1:2500), anti-AMPKα1 (2795, 1:2500) were purchased from Cell Signaling Technology. Anti-IRS1 (1:2500) and anti-phospho-IRS (Ser307) (07247, 1:2500) antibodies were purchased from Upstate Biotechnology. Anti-AMPKα1 (32047, 1:2500) was purchased from Abcam.

### Primary hepatocytes

Hepatocytes were isolated from liver of 12–14 weeks old L-Ago2 WT and L-Ago2 KO mice by a two-step perfusion method^55^ with a slight modification. Briefly, the liver was first perfused with 30 ml of HBSS supplemented with 10 mM HEPES, 0.5 mM EGTA and 5 mM glucose and then digested with 35 ml of Collagenase X (WAKO) at 100 U ml^−1^ dissolved in HBSS buffer supplemented with 10 mM HEPES and 5 mM CaCl_2_. Liver was collected after perfusion and hepatocyte were released and sedimented at 60 × *g* for 2 min. Hepatocyte suspension was then layered on a 40% percoll solution (GE Healthcare Life Sciences) and centrifuged at 800 × *g* for 10 min. The alive hepatocytes were recovered from the bottom of the tube and seeded on culture plates.

### Mouse embryonic fibroblasts

Ago2-deficient fibroblasts reconstituted with Ago2 WT or DA mutant that were kindly provided by Dr. Eric Lai^22^. We also generated *Ago2*^*fl/fl*^ MEFs through the 3T3 protocol and performed an adenovirus-mediated gene transfer for Cre or LacZ expression to obtain Ago2-deficient MEFs^56^. MEF cells were cultured in Dulbecco Modified Eagle Medium **(**DMEM) (Thermo Fisher Scientific: #11965) supplemented with 10% FBS. For western blot analyses of AMPK, MEF cells were plated at a density of 1 × 10^5^ cells per well of six-well plate for overnight. Next day, cells were kept under serum starvation condition in glucose-, pyruvate-, and glutamine-free DEME (Thermo Fisher Scientific: #A1443001).

### Quantitative real-time PCR analysis

For mRNA quantification, total RNA was extracted using Trizol reagent (Invitrogen). Total RNA was converted to first strand cDNA using SuperScript VILO™ cDNA Synthesis Kit (Invitrogen). Quantitative real-time PCR analysis was performed using SYBR Select Master Mix (Applied Biosystems) in a real-time PCR machine (QuantStudio 6 Flex Real-Time PCR system; Thermo Fisher Scientific). Primers are listed in Supplementary Table 4. To normalize expression data, β-actin mRNA was used as a housekeeping gene.

For miRNA quantification, total RNA was extracted using miRNeasy Micro Kit (Qiagen) according to manufacturer’s instructions. TaqMan miRNA assays (Life Technologies) were used and real-time PCR were carried out for mature miRNA quantification. Primary miRNAs were quantified using TaqMan Pri-miRNA assays. Sno202 and β-actin were used as internal controls.

### High-throughput sequencing of miRNA

Liver tissues were excised from mice, and stored in −80 °C after RNAlater (Invitrogen) treatment. Liver tissue was homogenized with QIAzol (Qiagen). Total RNA, including miRNA, was extracted using the miRNeasy Micro Kit (Qiagen). High-throughput sequencing of miRNA was processed according to TruSeq Small RNA Sample Preparation Guide (Illumina). Briefly, the total RNA was run on an agarose gel and the band corresponding to the size of miRNAs was cut out for further processing. Sequencing adapters were ligated to the size-selected RNA molecules, followed by reverse transcription to obtain the cDNA library, which was subsequently sequenced by Illumina HiSeq2500.

### Bioinformatic analysis of miRNA seq

Adapter sequences were removed with fastx_toolkit (v0.0.14, -a TGGAATTCTCGGGTGCCAAGG -l 15 -M 20 -c, http://hannonlab.cshl.edu/fastx_toolkit/) for NCD (Fig. 1a) and S961 (Fig. 3d) samples, and TrimGalore (v0.4.3, -e 0.1 -q 20 -O 1 -a AGATCGGAAGAGCACACGTC, https://www.bioinformatics.babraham.ac.uk/projects/trim_galore/), and Cutadapt (v1.9.1, http://cutadapt.readthedocs.io) for HFD samples (Fig. 6a), with reads that became shorter than 15 bases, remaining reads were filtered with the FASTX package (v0.0.14), using a quality threshold of 20 over at least 90% of the read. To get raw counts for each mature miRNA, miRExpress (v2.1.4, http://mirexpress.mbc.nctu.edu.tw/) was used to align and create expression count profiles with default parameters. The alignment step (alignmentSIMD) uses a miRNA precursor file (mmu_precursor.txt), supplied with the miRExpress v2.1.4 and the analysis step uses mmu_miRNA.txt with mature miRNA information for precursor sequences. Raw counts were normalized using DESeq2 (v1.18.0, http://bioconductor.org/packages/release/bioc/html/DESeq2.html), to compare mature miRNAs’ relative abundance. Differentially expressed miRNAs were predicted also using DESeq2. For NCD (Fig. 1a) and HFD samples (Fig. 6a), we applied |fold change| > 2x and adjusted *p* (FDR BH) < 0.05 as filters. For S961 (Fig. 3d), we applied |fold change| > 1.25x and *p* < 0.0005. Heatmaps were created by the pheatmap package (https://cran.r-project.org/web/packages/pheatmap/index.html).

### miRNA target pathway enrichment analysis

To predict the enriched target pathways, we used the mirPath web-server (v3, http://snf-515788.vm.okeanos.grnet.gr), based on DIANA-microT-CDS algorithm. We chose KEGG (http://www.genome.jp/kegg) database as a reference and *p* < 0.05 and MicroT threshold < 0.8 as filters to get significantly enriched KEGG pathways.

### Protein extraction and immunoblot analysis

To prepare protein lysates, cells were washed with cold PBS, followed by lysis in cold mammalian cell lysis buffer [MCLB: 50 mM Tris-HCl (pH 7.4), 150 mM NaCl, 10 mM NaF, 5% glycerol, 1% NP-40, 1% protease and phosphatase inhibitor cocktail]. After homogenization on ice, the cell lysates were centrifuged, and the supernatants were used for western blot analyses. For preparation of liver tissue lysates, the tissues were placed in a cold MCLB and homogenized on ice. The tissue lysates were centrifuged, and the supernatants were used for further experiments. Uncropped scans of immunoblots are shown in Supplementary Fig. 9.

### Morphological and immunohistochemical analysis of hepatic and pancreatic tissues

Liver and pancreas were taken, fixed with 10% formalin, and paraffin-embedded sections were prepared for further analysis. Paraffin sections were stained with H&E and Periodic Acid-Schiff (PAS) for morphology analyses. For the immunohistochemical staining, the following primary antibodies were used: guinea pig polyclonal anti-insulin (Abcam) and rabbit monoclonal anti-glucagon (Abcam). The following secondary antibodies were used: Alexa Fluor 488–conjugated AffiniPure Goat Anti-Guinea Pig (Jackson Immunoresearch) and Alexa Fluor 594–conjugated AffiniPure Goat Anti-rabbit IgG (Jackson Immunoresearch). The nuclei were stained using 4′,6-diamidino-2-phenylindole (DAPI), and sections were preserved using fluorescence mounting medium (Electron Microscopy Science). Images were acquired on a Nikon 90i Upright. ImageJ was used to process the images.

### Palmitate and acetate oxidation assays

Seahorse Bioscience XFe96 extracellular Flux Analyzers were used^53^ to detect palmitate oxidation in primary hepatocytes. Palmitate oxidation was measured by oxygen consumption rate (OCR) with modification. Primary hepatocytes were seeded at a density of 6000 cells per well of a XFe96 cell culture microplate and incubated in William E supplemented with 10% FBS. Next day, cells were cultured with DMEM (5.5 mM glucose) supplemented with 10% FBS and 2 mM Glutamax for 16 h. Then, these cells were incubated in DMEM (5.5 mM glucose) supplemented with 1% FBS, 1 mM Glutamax, and 0.5 mM carnitine for 2 h and then equilibrated for 1 h in palmitate oxidation assay medium (111 mM NaCl, 4.7 mM KCl, 1.25 mM CaCl_2_, 2 mM MgSO_4_, 1.2 mM NaH_2_PO_4_) supplemented with 2.5 mM glucose, 0.5 mM carnitine, and 5 mM HEPES at 37 °C for 1 h. 15 min prior to the assay, additional 400 μM of Etomoxir or vehicle was added. Palmitate-BSA or BSA was added to the microplate just prior to starting the assay. Sequential injections of 2 μM oligomycin, 2 μM phenylhydrazone, and 1 μM rotenone/1 μM antimycin A1 were used to examine mitochondrial oxidative status.

For acetate oxidation assay, sodium acetate (1 M, pH 7.4) was prepared followed by filtering. Sequential injection of 5 mM acetate, 2 μM oligomycin, 2 μM phenylhydrazone and 1 μM rotenone/1 μM antimycin A1 were used to examine mitochondrial oxidative status. Each readout was normalized to total cellular protein levels.

### Pyruvate oxidation assay and glycolysis stress test

Primary hepatocytes were isolated and seeded at a density of 6000 cells per well of a XFe96 cell culture microplate and incubated in William E supplemented with 10% FBS. Next day, cells were cultured with DMEM (5.5 mM glucose) supplemented with 10% FBS. Next day, prior to performing an assay, growth medium in the wells of XF cell plate was exchanged with 175 μl of XF base medium (pH 7.4) containing no exogenous fuel substrate supplementation (Seahorse Bioscience) at 37 °C for 1 h. Sequential injection of 2 mM pyruvate, 2 μM oligomycin, 2 μM phenylhydrazone, and 1 μM rotenone/1 μM antimycin A1 were used to examine mitochondrial oxidative status.

For glycolysis stress test, sequential injection of 10 mM glucose, 2 μM oligomycin, and 2-DG (2-deoxy-glycose, a glucose analog) were used to examine glycolysis stress. Each readout was normalized to total proteins.

### ATP/ADP ratio assay

The ATP/ADP ratio in the mouse liver extract or primary hepatocytes was determined using a bioluminescent ATP/ADP Ratio Assay Kit (Abcam) according to the manufacturer’s instructions. For mouse liver samples, the samples were immediately frozen in liquid nitrogen and powdered with a mortar. Tissue powders were suspended in the provided lysis buffer (10 μl mg^−1^ of tissue powder) for 5 min at room temperature, followed by centrifugation at 10,000 × *g* for 1 min to pellet insoluble material. For mouse primary hepatocytes, cells were plated at a density of 0.8 × 10^5^ cells per well of 24-well plate for overnight. Next day, cells were cultured with XF base media or DMEM no glucose and glutamine media (Gibco: A1443001) in the presence or absence of 5 mM Sodium Palmitate without FBS for 2 h. Data were normalized by the amount of protein present in the supernatant.

### ATP and ADP assays

The ATP or ADP in the liver extract was determined using a ATP Assay Kit (Abcam) or ADP Assay Kit (Abcam), respectively, according to the manufacturer’s instructions. Data were normalized by the amount of protein present in the supernatant.

### Protein synthesis analysis

Click-iT labeling technology was used for the detection of nascent protein synthesis in cells according to manufacturer’s instructions (Thermo Fisher Scientific). Mouse primary hepatocytes were seeded at 0.6 × 10^6^ cells per well in a six-well plate. Cells were then incubated in methionine- and cysteine-free DMEM containing 25 μM of azide-linked methionine analog AHA in the presence or absence of 200 μM phenformin or 10 μM Rotenone for 5 h. Azide-labeled protein lysate from harvested cells was determined by using Click-iT® TAMRA Protein Analysis Kit according to manufacturer’s instructions (Thermo Fisher Scientific) and Typhoon FLA9500 scanner (GE Healthcare) with the excitation at 532 nm. Coomassie Brilliant Blue (CBB)-based staining (Thermo Fisher Scientific; GelCode Blue) for total protein served as a loading control.

### Mitochondrial DNA copy number

Total DNA were purified from mouse liver using GeneJet Genomic DNA purification kit according to the manufacturer’s instruction (Thermo Fisher Scientific). Mitochondrial DNA copy number was detected by qPCR^57^.

### Glucose production assay

Mouse primary hepatocytes were cultured in 12-well plates (0.4 × 10^6^ cells per well) in William E supplemented with 10% FBS. Next day, cells were cultured with 1 ml of DMEM (5.5 mM glucose) supplemented with 10% FBS. Post plating for 21 h, cells were washed twice with PBS and were subjected 3–4 h to serum starvation with FBS-free DMEM (5.5 mM glucose). After washing twice with PBS, cells were cultured in 0.4 ml of glucose production buffer consisting of glucose-free DMEM (pH 7.4) without phenol red supplemented with 20 mM sodium lactate, 2 mM sodium pyruvate, 2 mM l-glutamine and 15 mM HEPES^58^. Cells were incubated at 37 °C for 4.5 h with or without Bt-cAMP or pCPT-cAMP. Both medium and cells were collected. The glucose concentration was measured with the Autokit Glucose (WAKO) and was normalized by the total protein content.

### Mitochondrial isolation

Liver tissue samples were minced and kept in ice-cold PBS containing proteinase inhibitor immediately after harvest. Tissues were then homogenized in resuspension buffer (RSB)/EDTA (10 mM Tris pH 6.7, 10 mM NaCl, 0.1 mM EDTA pH 8.0) containing proteinase inhibitor. The homogenized samples were filtered through 30-μm filter and sucrose concentration was then adjusted to 250 mM by adding 2 M sucrose. The suspension was centrifuged at 540 G for 3 min at 4 °C and the supernatant was collected for further separation. Crude mitochondrial for functional analysis were sedimented from the supernatant by centrifugation at 9650 × *g* for 10 min at 4 °C. To prepare pure mitochondria, the crude mitochondria were resuspended in ice-cold separation buffer, mixed with anti-TOM22 MicroBeads and enriched on a MACS column (Miltenyi Biotec). Magnetically purified mitochondria were incubated with 100 μg ml^−1^ of RNase for 30 min on ice and 10× volume of T10E20/sucrose was used to wash the mitochondria. Isolated mitochondria were pelleted and kept in −80 °C until use.

### Luciferase assay

Luciferase plasmids harboring the Ampka1 3′ UTR were generated as follows. Ampka1 3′ UTR were amplified by using primer: 5′-CCCA*GAATTC*CATTTAAGTTACAGCCTG-3′ and 5′-GCAT*CTCGAG*GTTCCTTTCATGAGAAATCAAC-3′, and cloned into EcoRI and XhoI restriction enzyme sites of pEZX-MT06 (GeneCopoeia). The EcoRI and XhoI sites are shown in italics. PCR was performed using Phusion High-Fidelity DNA polymerase (New England BioLabs). Mutagenesis of the 3′ UTR was performed with the QuickChange Lightining Site-Directed Mutagenesis Kit (Agilent Technologies) according to the manufacturer’s instructions. Primer sequences used to mutate the miR-148/miR-152 binding site in the Ampka1 are the following: 5′-CATGATAGCTTGCATAAAAGATGACGCTATAGTTTA**ACGT**CTGATTTCCGGACAAAAATG-3′ and 5′-CATTTTTGTCCGGAAATCAG**ACGT**TAAACTATAGCGTCATCTTTTATGCAAGCTATCATG-3′. The mutated residues are indicated in bold.

Mouse primary hepatocytes were plated at a density of 0.8 × 10^5^ cells per well of 24-well plate, and transfected with 0.5 μg of each control (pEZX-MT06), Ampka1 (Prkaa1) (MmiT024101-MT06) and Ampka1 mutant using Lipofectamine 3000 (Life Technologies) according to the manufacturer’s instructions. The transfected cells were incubated with DMEM (5.5 mM glucose) with 10% FBS media for 8 h. Luciferase activities were measured with Luc-Pair Duo-Luciferase Assay Kit 2.0 (GeneCopoeia) and GLOMAX 96 Microplate Luminometer (Promega).

### Polysome profiling

Preparations of cellular extracts for polysome profiles, sucrose gradient centrifugation, and profile recording^59^. Heparin was omitted from lysates used to analyze *Ampka1*, *Cs*, and *β-actin* mRNAs by qRT-PCR due to its inhibitory effect on the PCR. Sucrose gradient fractions were collected by upward displacement, and 50 pg of synthetic luciferase mRNA (Promega) and 15 μg of GlycoBlue (Life Technologies) were added to each fraction to control for extraction and PCR efficiency and to improve RNA recovery, respectively. After extraction and precipitation of RNA from sucrose fractions^59^, precipitated RNA was washed twice with ice-cold 70% ethanol, dried, and resuspended in RNase-free H_2_O. Equal volumes of RNA from each fraction were subjected to cDNA synthesis and qRT-PCR analysis. Levels of *Ampka1*, *Cs*, and *β-actin* mRNAs in each fraction were normalized to luciferase mRNA and plotted as the percentage of total mRNAs from all 12 fractions.

### Statistical analysis

Experimental results were shown as the mean ± SEM. The mean values for biochemical data from each group were compared by Student’s *t*-test. Comparisons between multiple time points were analyzed using repeated-measures analysis of variance, two-way ANOVA. Analyses of miRNA sequencing were done by DESeq2^60^.

### Data availability

The RNA-Seq data generated in this study have been deposited in the NCBI Sequence Read Archive (SRA, http://www.ncbi.nlm.nih.gov/sra) under the BioProject ID PRJNA395686. The accession codes are SAMN07415658, SAMN07415659, SAMN07415660, SAMN07415661, SAMN07415662, and SAMN07415663 for the NCD study, SAMN07415764, SAMN07415766, SAMN07415768, SAMN07416029, SAMN07415767, SAMN07415769, SAMN07415765, SAMN0741630, SAMN07416036, SAMN07416037, SAMN07416038, and SAMN07416039 for the S961 study, and SAMN07413900, SAMN07413901, SAMN07413906, SAMN07413907, SAMN07413949, and SAMN07413951 for the HFD study. All other data supporting the findings of this study are available from the corresponding author on reasonable request.

## Electronic supplementary material

Supplementary Information
