# Supplementary material for: Hepatic Ago2-mediated RNA silencing controls energy metabolism linked to AMPK activation and obesity-associated pathophysiology
Source: Nat Commun. 2018 Sep 10;9:3658. doi: 10.1038/s41467-018-05870-6 (PMC6131149; doi:10.1038/s41467-018-05870-6)
Supplement: Supplementary file 1 — Supplementary Information [file 41467_2018_5870_MOESM1_ESM.pdf]

Supplementary Information

Hepatic Ago2-mediated RNA silencing controls energy metabolism linked to AMPK activation and obesity-associated pathophysiology

Zhang et al.

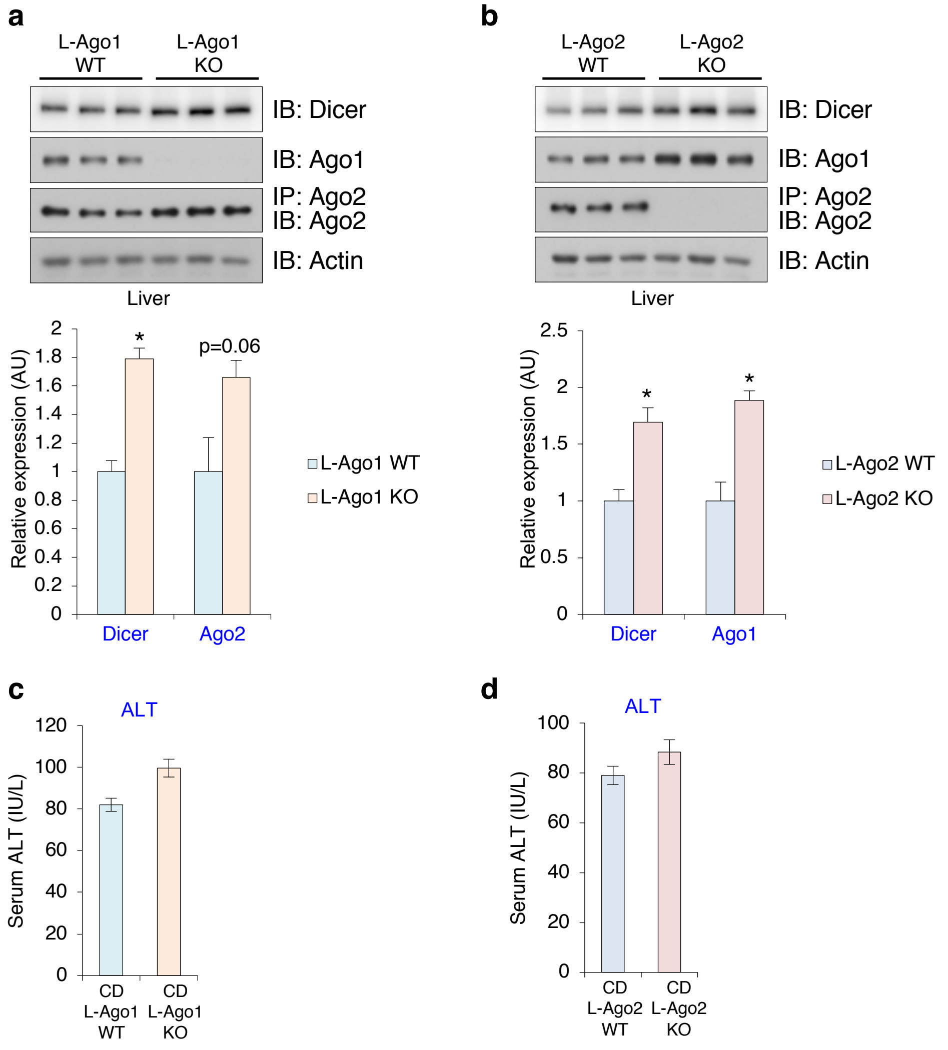

Supplementary Figure 1 | Generation of L-Ago1 KO and L-Ago2 KO mice.
(a and b) Western blot analyses of Ago1, Ago2, and Dicer in the liver of L-Ago1 WT (n=3) and L-Ago1 KO (n=3) (a) and L-Ago2 WT (n=3) and L-Ago2 KO (n=3) (b) mice fed NCD at 25 weeks of age. The graphs below show the quantification of the results. (c and d) Serum ALT levels of L-Ago1 WT (n=7), L-Ago1 KO (n=4), L-Ago2 WT (n=5), and L-Ago2 KO (n=6) mice fed NCD at 9 weeks of age. Data are shown as the mean ± SEM. $^{*}p<0.05$

Supplementary Fig. 2

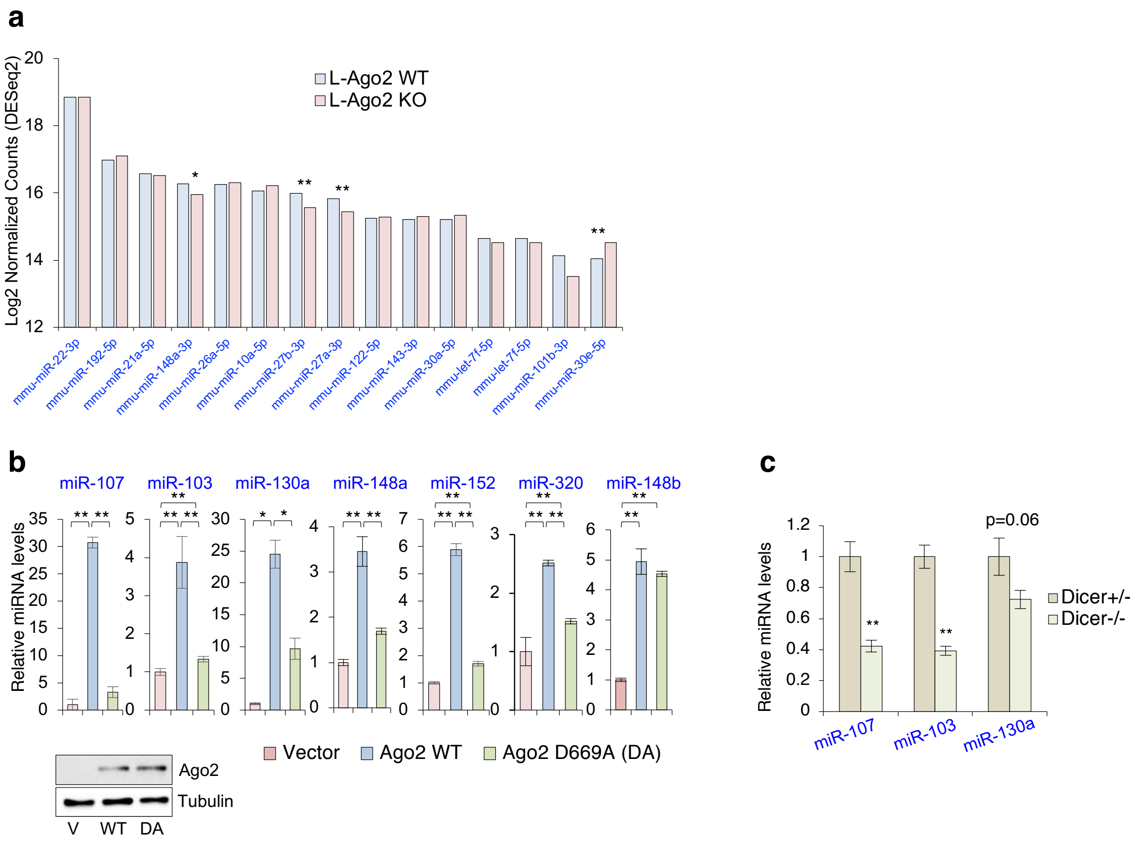

d

miRNA significantly reduced in L-Ago2 KO	Point 1 (Small loop size)	Point 2 (matching at center)	Point 3 (Long stem)	Point 4 (no 3' overhang)	Total points
mmu-miR-101a-3p	0	1	0	1	2
mmu-miR-101a-5p	0	1	0	1	2
mmu-miR-101b-3p	1	1	1	1	4
mmu-miR-103-1-3p	1	1	1	1	4
mmu-miR-107-3p	1	1	1	0	3
mmu-miR-130a-3p	1	1	0	0	2
mmu-miR-140-3p	1	1	1	1	4
mmu-miR-141-3p	1	1	1	1	4
mmu-miR-148a-3p	1	1	1	1	4
mmu-miR-148b-3p	1	1	1	1	4
mmu-miR-17-5p	1	1	1	1	4
mmu-miR-1839-5p	1	1	0	0	2
mmu-miR-185-5p	1	1	1	1	4
mmu-miR-194-1-5p	1	1	1	1	4
mmu-miR-1981-3p	1	1	1	0	3
mmu-miR-27a-3p	0	1	1	1	3
mmu-miR-27b-3p	1	1	1	1	4
mmu-miR-29b-2-3p	0	1	1	1	3
mmu-miR-320-3p	1	1	1	1	4
mmu-miR-365-2-3p	1	1	1	1	4
mmu-miR-425-5p	1	1	1	1	4
mmu-miR-802-3p	1	1	1	0	3
mmu-miR-802-5p	1	1	1	0	3
mmu-miR-93-5p	1	1	1	1	4

e

Total Points	miRNA reduced in L-Ago2 KO (Number)	miRNA reduced in L-Ago2 KO (%)
0	0	0.0
1	0	0.0
2	4	16.7
3	6	25.0
4	14	58.3

f

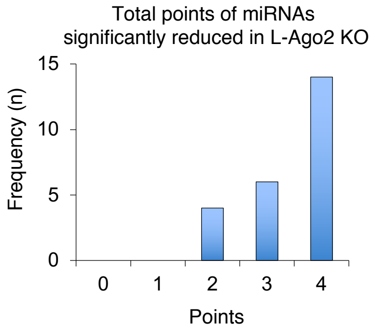

Supplementary Figure 2 | Analyses of miRNAs regulated by Ago2 in the liver.
(a) Raw counts of miRNA Seq were normalized using DESeq2 and transformed by Log2 to show top 15 miRNAs in livers of L-Ago2 WT and L-Ago2 KO mice fed NCD at 9 weeks of age. The most abundant miRNAs (top-15) are presented. Averaged normalized counts of significant miRNAs were plotted to show their relative abundances. *P*-values between two groups (* < 0.01, ** < 0.001, DESeq2 test) are indicated on the top of bar plots. (b) The effect of Ago2 WT or mutant reconstitution on MD-miRNAs expression in Ago2-deficient MEFs. Ago2-deficient MEFs were reconstituted with Ago2 variants. A panel below shows expression levels of Ago2 variants analyzed by western blotting. V: vector, WT: Ago2 WT, DA: Ago2 D669A mutant. Data are shown as the mean $\pm$ SEM. **p*<0.05, ***p*<0.01. (c) Expression levels of MD-miRNAs were assessed in Dicer$^{+/-}$ and Dicer$^{-/-}$ MEFs. Data are shown as the mean $\pm$ SEM. ***p*<0.01.(d) List of miRNAs whose expression levels are statistically reduced in the liver of L-Ago2 KO compared L-Ago2 WT mice and their structure analysis. There are mainly four proposed characteristics of miRNAs processed by Ago2 as follows: 1) loop size is less than 10 nt, 2) perfect matching at position 10 and 11 between guide and passenger strands, 3) long stem length (distance from 3′ to the loop is more than 30 nt), and 4) no 3′ hang out. These characteristics were evaluated to be scored as listed. (e and f) Proportion (e) and frequency (f) of miRNAs significantly reduced in the liver of L-Ago2 KO mice with different total points.

Supplementary Fig. 3

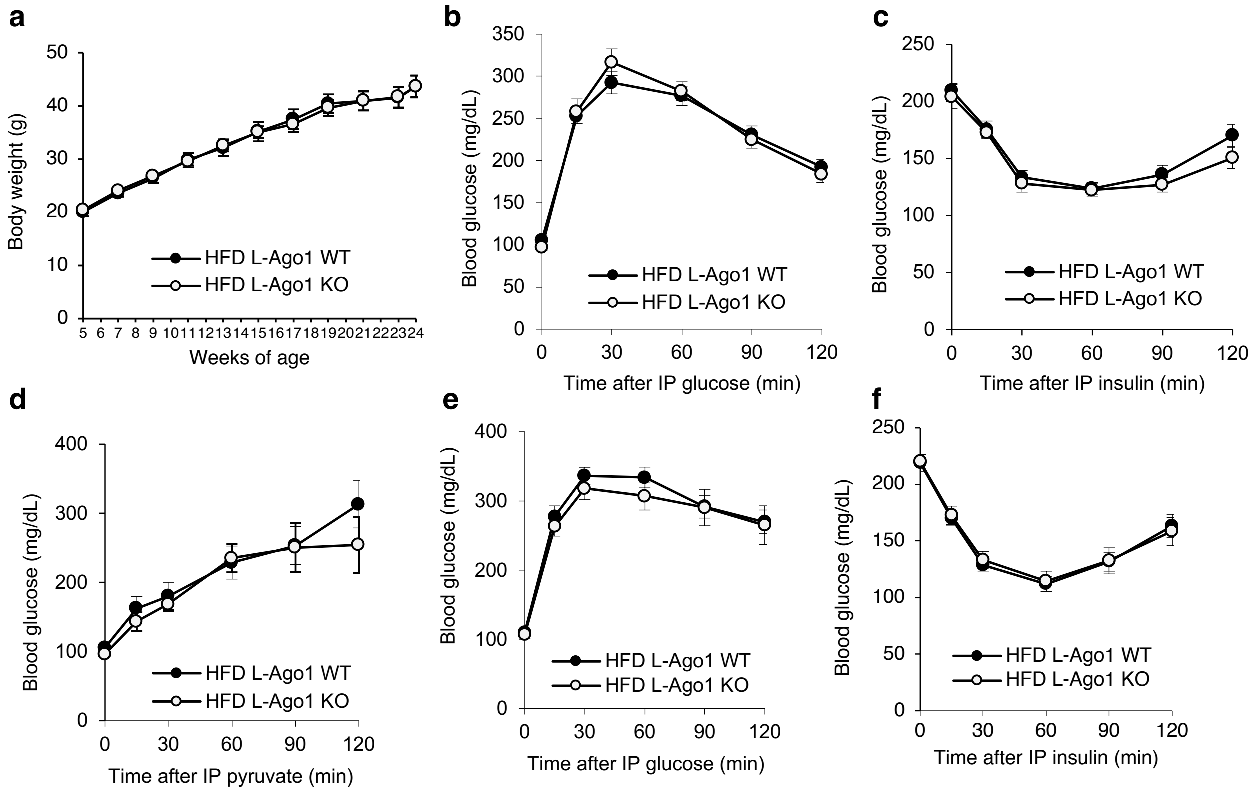

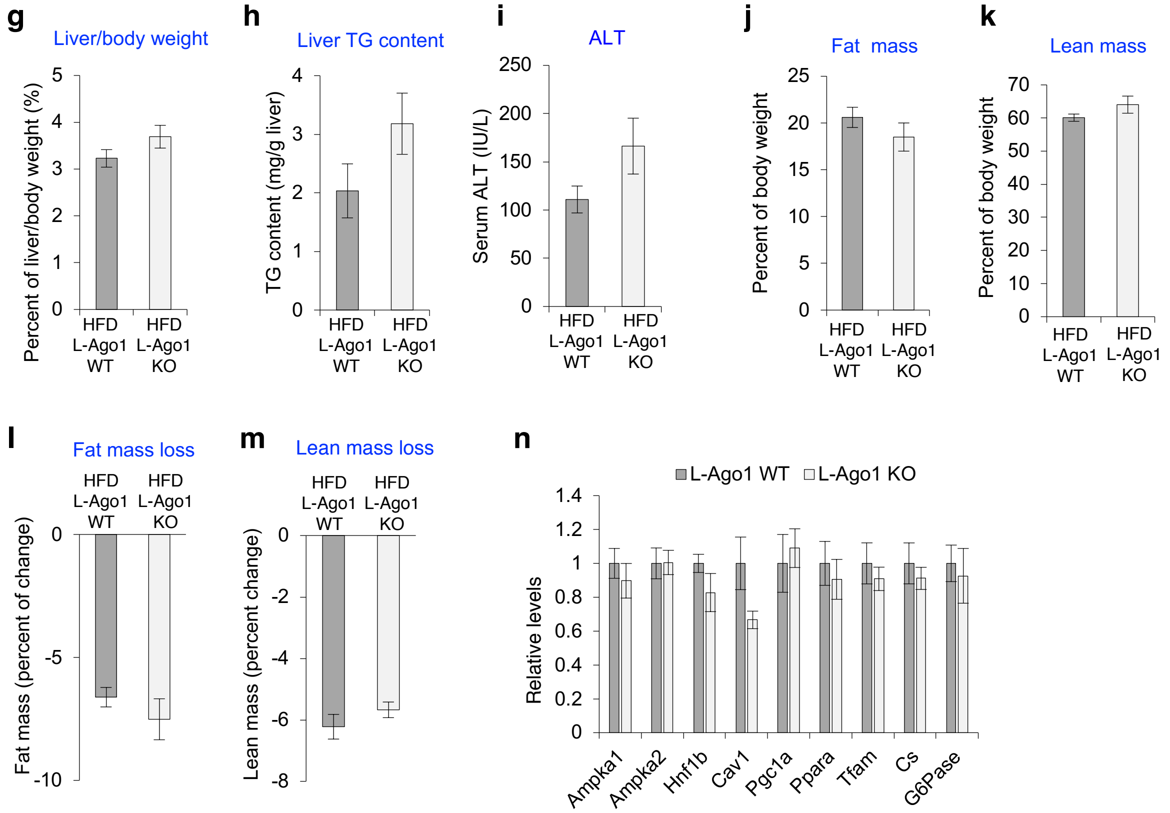

Supplementary Figure 3 | Effects of hepatic Ago1-deficiency on metabolism on HFD.
(a) Body weights of L-Ago1 WT (n=8) and KO (n=8) mice fed HFD, starting at 4 weeks of age. (b) Glucose tolerance test of L-Ago1 WT (n=17) and L-Ago1 KO (n=15) mice fed HFD at 13 weeks of age. (c) Insulin tolerance test of L-Ago1 WT (n=17) and L-Ago1 KO (n=15) mice fed HFD at 14 weeks of age. (d) Pyruvate tolerance test of L-Ago1 WT (n=8) and L-Ago1 KO (n=8) mice fed HFD at 17 weeks of age. (e) Glucose tolerance test of L-Ago1 WT (n=16) and L-Ago1 KO (n=15) mice fed HFD at 20 weeks of age. (f) Insulin tolerance test of L-Ago1 WT (n=16) and L-Ago1 KO (n=15) mice fed HFD at 21 weeks of age. (g-i) Percent of liver weight per body weight (g), liver triglyceride contents (h), and serum ALT levels (i) in L-Ago1 WT (n=8) and KO (n=8) mice fed HFD at 23 weeks of age. (j and k) Fat (j) and lean mass (k) as a percentage of body weight in L-Ago1 WT (n=8) and KO (n=8) mice fed HFD at 20 weeks of age. (l and m) Effects of a 14-hour fast on fat mass (l) and lean body mass (m) in L-Ago1 WT (n=7) and KO (n=7) mice fed HFD at 24 weeks of age. (n) Expression analyses of genes regulating energy metabolism in the liver of L-Ago1 WT (n=5) and L-Ago1 KO (n=5) fed HFD for 19 weeks.

Supplementary Fig. 4

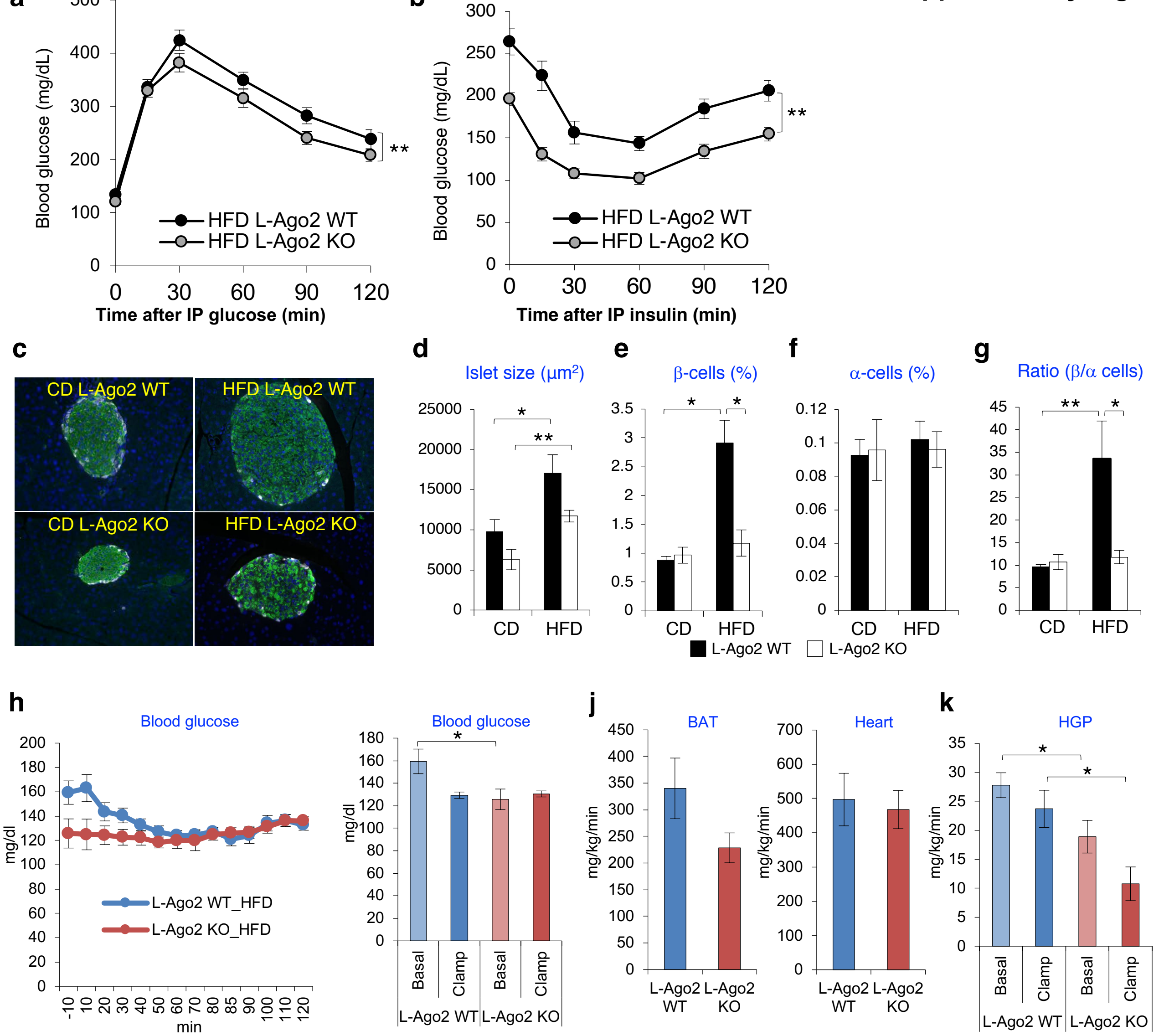

Supplementary Figure 4 | Ago2-deficiency in the liver improves systemic glucose metabolism.

(a) Glucose tolerance test performed in L-Ago2 WT (n=17) and KO (n=18) mice fed HFD at 13 weeks of age. (b) Insulin tolerance test of L-Ago2 WT (n=16) and KO (n=17) mice fed HFD at 21 weeks of age. (c) Immunohistochemical staining of insulin (green), glucagon (white) and DAPI (blue) in paraffin section (5 μm) of mouse pancreas. (d-g) Mean islet size (d), quantification of percentage of insulin-stained area (e), glucagon-stained area (f) in full pancreas section area, and stained insulin to glucagon ratio (g), collected from L-Ago2 WT (n=7) and L-Ago2 KO (n=7) mice fed HFD and L-Ago2 WT (n=5) and L-Ago2 KO (n=4) mice fed CD at 33 weeks of age. (h-k) Hyperinsulinemic-euglycemic clamp studies performed in L-Ago2 WT (n=6) and L-Ago2 KO (n=10) mice fed HFD at 24 weeks of age. (h) blood glucose levels throughout the clamp procedures. The graph on the right shows averages of blood glucose levels. (j) Tissue glucose uptakes in brown fat (BAT) and heart. (k) Hepatic glucose production (HGP) during the clamp. The graphs show the quantification of the results. Data are shown as the mean ± SEM. *p<0.05, **p<0.01.

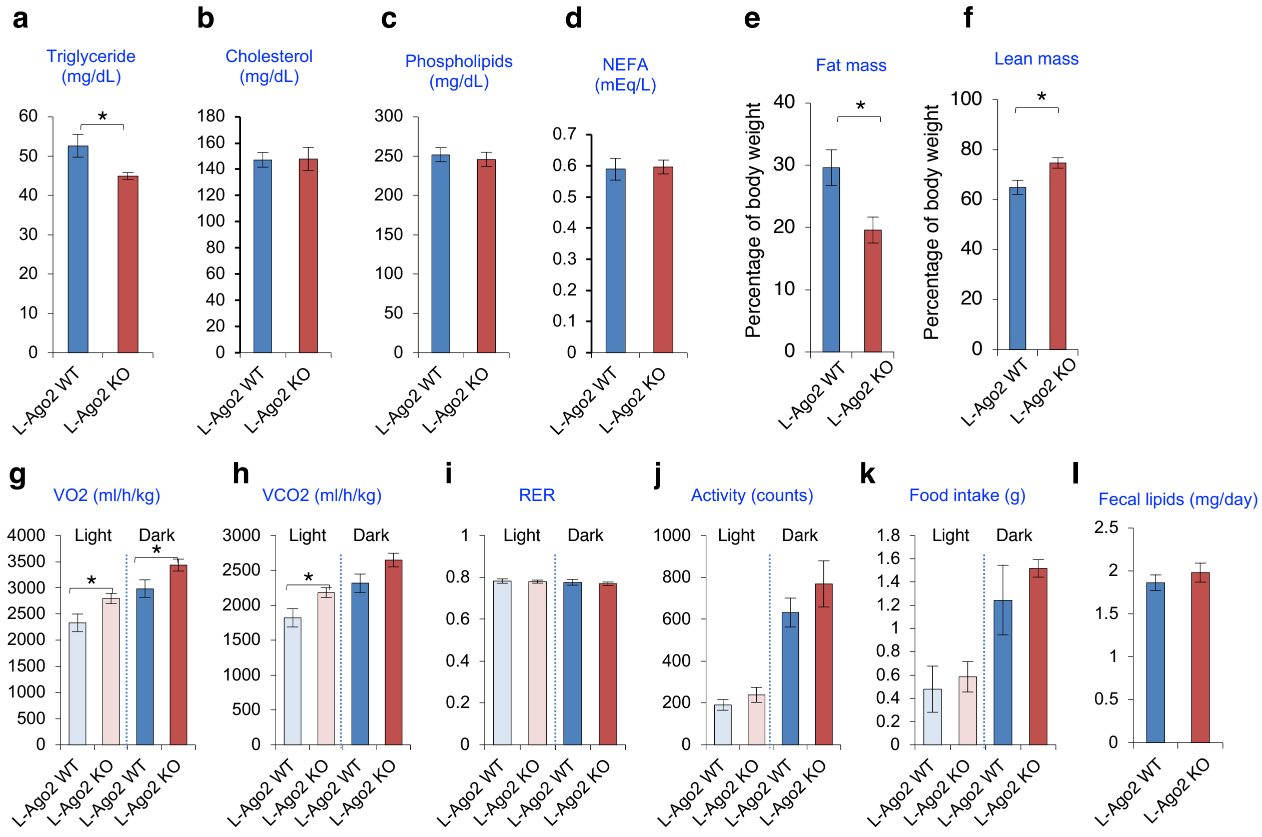

Supplementary Figure 5 | Effects of hepatic Ago2 deficiency on energy metabolism. (a-d) Plasma triglyceride (a), cholesterol (b), phospolipids (c), and NEFA (d) levels in L-Ago2 WT (n=8) and L-Ago2 KO (n=6) mice fed HFD at 15 weeks of age. (e and f) Fat (e) and lean mass (f) as a percentage of body weight of L-Ago2 WT (n=7) and L-Ago2 KO (n=7) mice fed HFD at 20 weeks of age (g-k) VO2 (g), VCO2 (h), RER (i) Activity (j), and food intake (k) measured by TSE PhenoMaster system in L-Ago2 WT (n=8) and L-Ago2 KO (n=8) mice fed HFD at 12weeks of age. (l) Fecal lipids excretion in L-Ago2 WT (n=8) and L-Ago2 KO (n=8) mice fed HFD at 18 weeks of age. Data are shown as the mean ± SEM. *$p<0.05$.

Supplementary Fig. 6

a

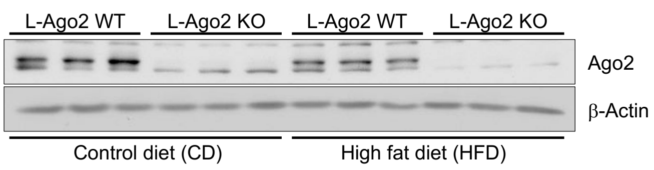

b

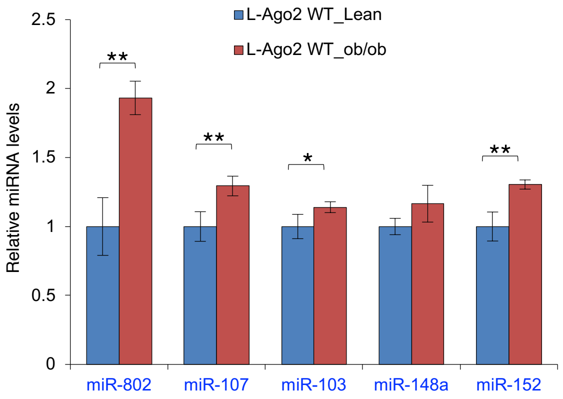

c

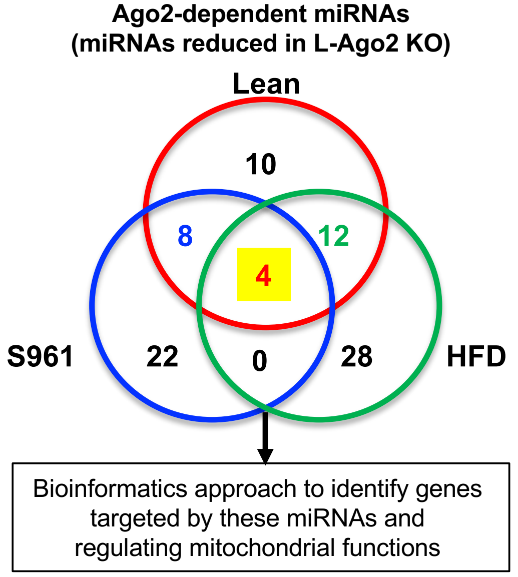

d

Lean	S961	HFD
**mmu-miR-148a-3p**	mmu-miR-1948-3p	**mmu-miR-101b-3p**
**mmu-miR-148b-3p**	mmu-miR-222-3p	mmu-miR-365-3p
mmu-miR-101a-3p	**mmu-miR-320-3p**	**mmu-miR-148b-3p**
**mmu-miR-802-3p**	mmu-miR-421-3p	**mmu-miR-107-3p**
**mmu-miR-802-5p**	mmu-miR-186-5p	**mmu-miR-194-5p**
**mmu-miR-101b-3p**	**mmu-miR-365-3p**	mmu-miR-30c-5p
**mmu-miR-27a-3p**	mmu-miR-1981-5p	mmu-miR-378c
**mmu-miR-27b-3p**	**mmu-miR-103-3p**	**mmu-miR-185-5p**
**mmu-miR-425-5p**	**mmu-miR-107-3p**	mmu-miR-744-5p
mmu-miR-140-3p	mmu-miR-1839-3p	mmu-miR-149-5p
**mmu-miR-93-5p**	mmu-miR-574-5p	mmu-miR-378a-3p
**mmu-miR-130a-3p**	mmu-miR-6964-3p	mmu-miR-152-3p
mmu-miR-29b-3p	mmu-miR-32-5p	mmu-miR-221-3p
**mmu-miR-185-5p**	**mmu-miR-93-5p**	**mmu-miR-802-5p**
**mmu-miR-148b-5p**	mmu-miR-292b-5p	**mmu-miR-1839-5p**
**mmu-miR-1839-5p**	mmu-miR-339-5p	mmu-miR-23b-3p
**mmu-miR-194-5p**	mmu-miR-330-5p	**mmu-miR-148b-5p**
**mmu-miR-365-3p**	mmu-miR-18a-5p	mmu-miR-122-5p
**mmu-miR-103-3p**	**mmu-miR-802-3p**	mmu-miR-16-5p
**mmu-miR-107-3p**	**mmu-miR-802-5p**	**mmu-miR-27b-3p**
**mmu-miR-17-5p**	mmu-miR-193a-3p	**mmu-miR-103-3p**
**mmu-miR-1981-3p**	mmu-miR-7219-3p	**mmu-miR-148a-3p**
**mmu-miR-320-3p**	mmu-miR-107-5p	mmu-miR-98-5p
mmu-miR-101a-5p	mmu-miR-6978-3p	**mmu-miR-22-5p**
mmu-miR-141-3p	mmu-miR-17-3p	mmu-miR-146b-5p
mmu-miR-200b-3p	mmu-miR-3105-3p	mmu-miR-199b-5p
**mmu-miR-148a-5p**	mmu-miR-128-3p	mmu-miR-100-5p
**mmu-miR-181a-5p**	**mmu-miR-130a-3p**	mmu-let-7i-5p
mmu-miR-30e-5p	mmu-miR-484	**mmu-miR-27a-3p**
**mmu-miR-125b-5p**	**mmu-miR-17-5p**	mmu-miR-214-3p
mmu-miR-150-5p	**mmu-miR-194-5p**	mmu-miR-199a-3p
mmu-miR-411-5p	**mmu-miR-425-5p**	mmu-miR-199b-3p
mmu-miR-122-3p	**mmu-miR-1981-3p**	**mmu-miR-125b-5p**
**mmu-miR-22-5p**	mmu-miR-33-5p	mmu-miR-142a-3p
		mmu-miR-199a-5p
		mmu-miR-146a-5p
		mmu-miR-10a-5p
		mmu-miR-342-3p
		mmu-miR-10b-5p
		**mmu-miR-181a-5p**
		mmu-miR-143-3p
		mmu-miR-214-5p
		mmu-miR-592-5p
		**mmu-miR-148a-5p**

Supplementary Fig. 6
(continued)

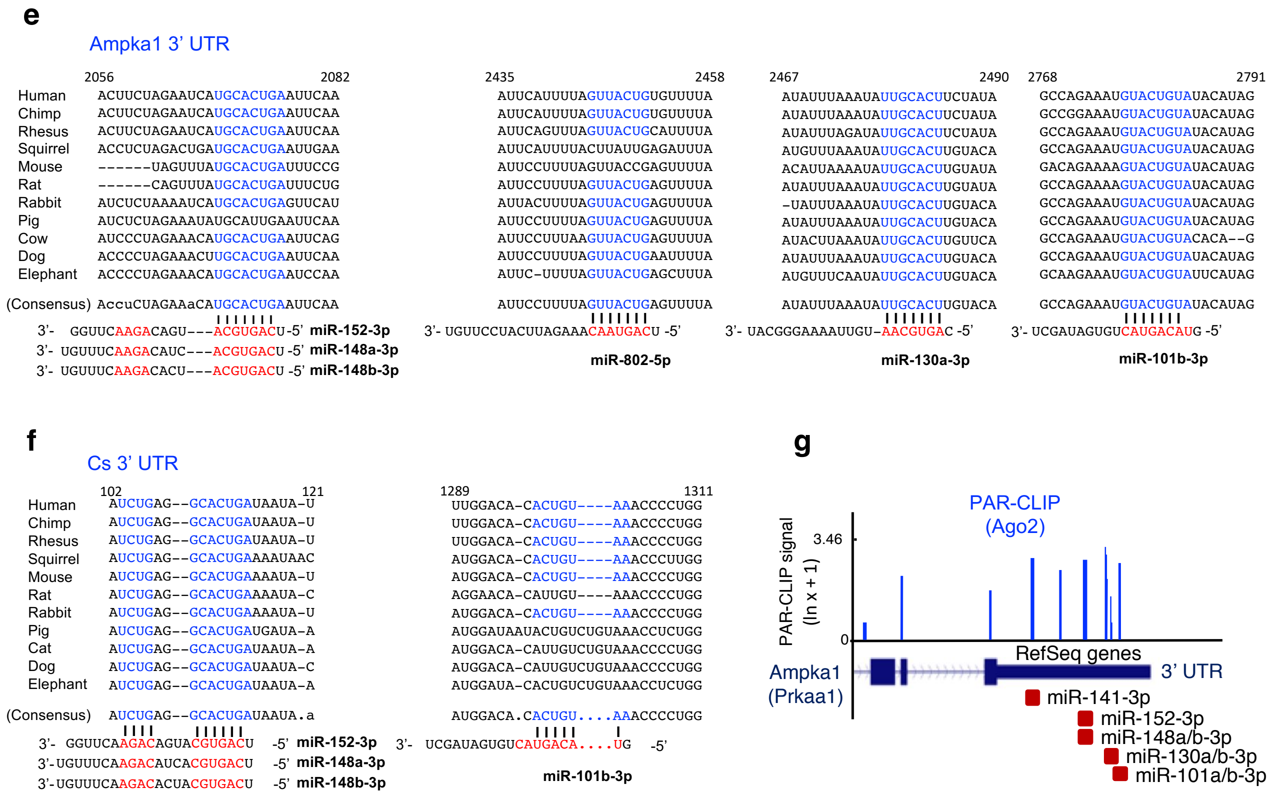

Supplementary Figure 6 | Identification of Ago2-dependent miRNAs and their targets.
(a) Western blot analyses of Ago2 in livers of L-Ago2 WT and KO mice fed CD or HFD at 30 weeks of age. (b) Expression levels of specific MD-miRNAs in livers *ob/ob* mice (n=7) and their lean controls (n=5). Data are shown as the mean ± SEM. **p*<0.05, ***p*<0.01. (c) Venn diagram showing miRNAs reduced in livers of L-Ago2 KO compared to L-Ago2 WT fed CD, fed HFD, and treated with S961. (d) Lists of miRNAs whose levels are significantly downregulated in livers of Ago2 KO mice in lean, HFD-feeding, and S961 treated conditions. (e and f) Potential binding sites of Ago2-dependent miRNAs in the 3′ UTR of *Ampka1* (e) and *Cs* (f) showing species conservation. (g) Ago2 PAR-CLIP analysis of mouse bone marrow-derived macrophage. Ago2 PAR-CLIP reads, shown in blue, overlap with multiple distinct, high-confidence miRNA binding sites. The *Ampka1 (Prkaa1)* transcript is indicated by blue boxes (the wider box indicates the coding region and the narrower box indicates the 3′ UTR). RefSeq, reference sequence database. TargetScan 7.1 was used to identify potential binding sites for miRNAs whose expression levels changed in the Ago2-deficient conditions.

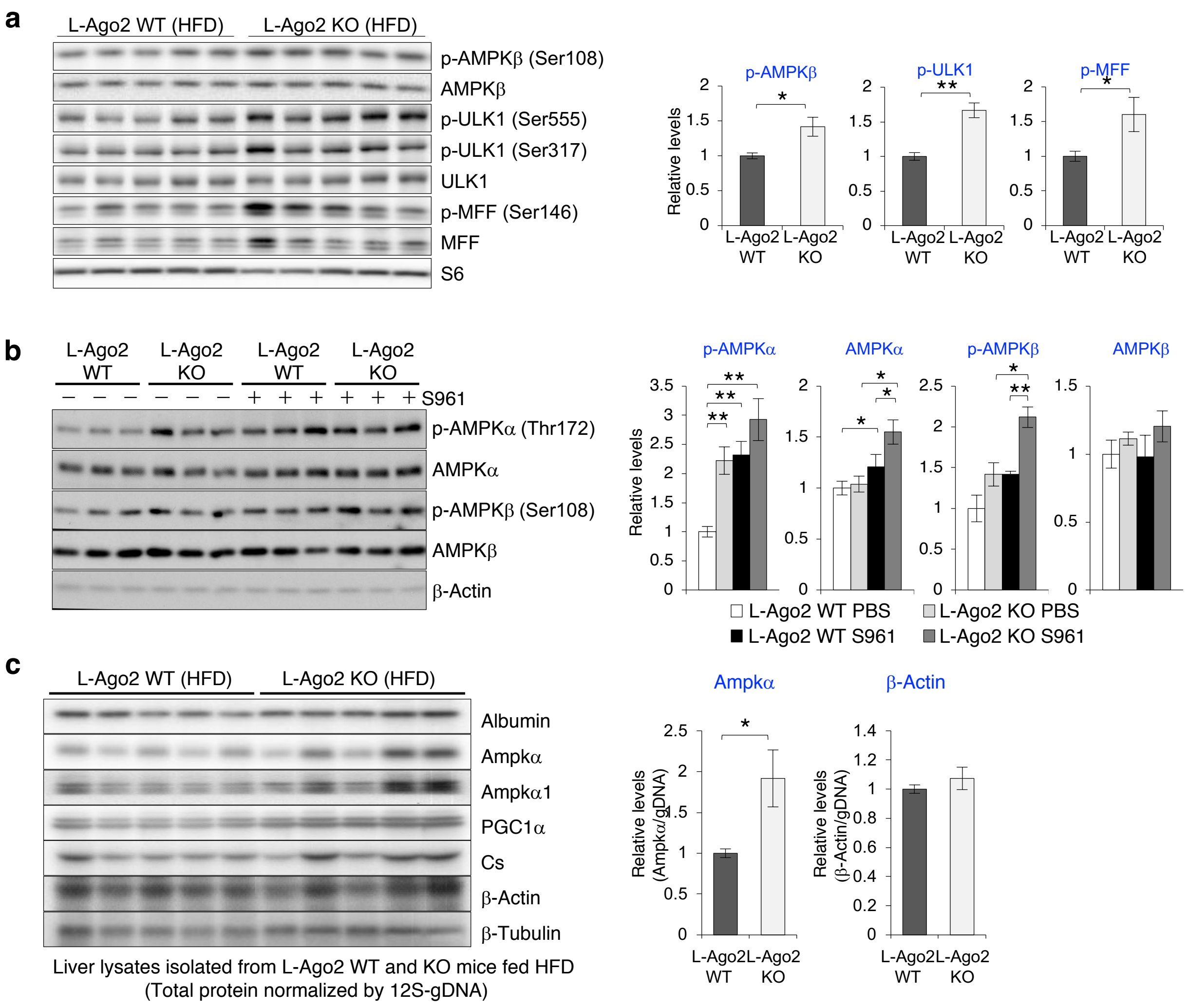

Supplementary Figure 7 | Enhanced activation of the AMPK pathway in L-Ago2 KO mice. (a) Western blot analyses of the liver lysates from L-Ago2 WT (n=5) and KO (n=5) mice fed HFD at 25 weeks of age. (b) Western blot analyses of the AMPK pathway in livers of L-Ago2 WT and L-Ago2 KO treated with S961 for 2 weeks. (c) Western blot analysis of total and specific protein levels normalized by 12S-genomic DNA in the liver of L-Ago2 WT (n=5) and KO (n=5) mice fed HFD at 30 weeks of age. The graphs show the quantification of the results. Data are shown as the mean ± SEM. *p<0.05, **p<0.01.

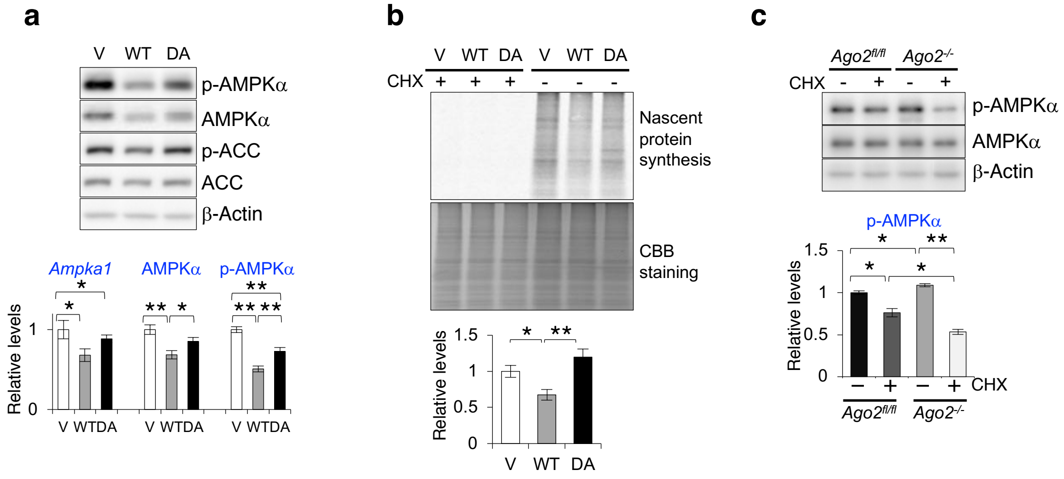

Supplementary Figure 8 | Ago2-deficiency enhances energy expenditure linked AMPK activation and protein translation in MEFs.

(a and b) Effect of Ago2 WT (n=5) or slicer activity-defect Ago2 mutant (DA) (n=5) reconstitution on expression of Ampka1 mRNA and AMPK protein, and activation of AMPK (a) in Ago2-deficient MEFs (n=5), and nascent protein synthesis (b) in Ago2-deficient MEFs (n=4, n=4, n=4 for Vector, WT, DA, respectively). (c) AMPK activation in Ago2 WT (n=3) and KO (n=3) MEFs in the presence or absence of 25 μg/ml cycloheximide (CHX) for 8 hours. Data are shown as the mean ± SEM. *p<0.05, **p<0.01.

Supplementary Figure 9 | Uncropped Western blots.
Dot line boxes indicate the cropped areas shown in the figures.

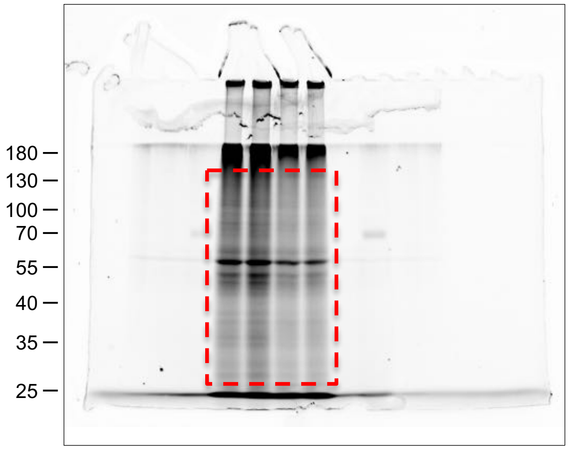

Nascent Protein Synthesis

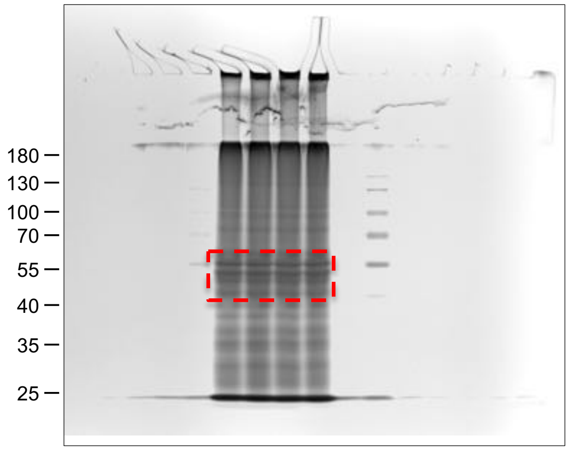

CBB staining

Fig. 6h (Phenformin)

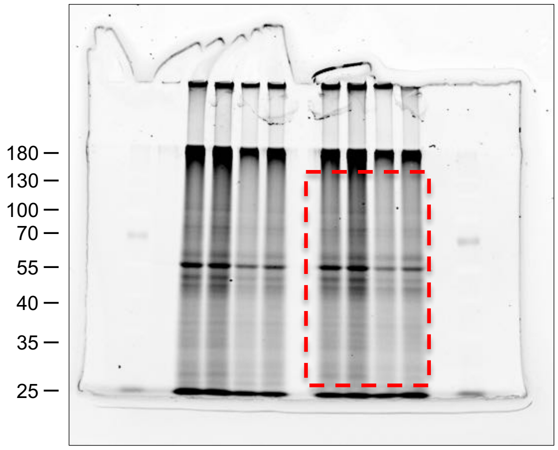

Nascent Protein Synthesis

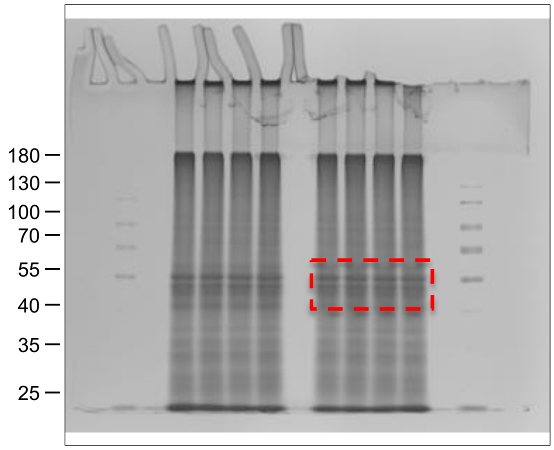

CBB staining

Fig. 6h (Rotenone)

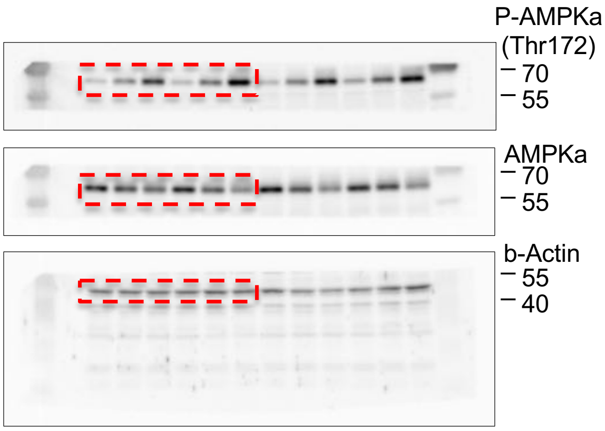

Fig. 6g

Supplementary Fig. 9
(continued)

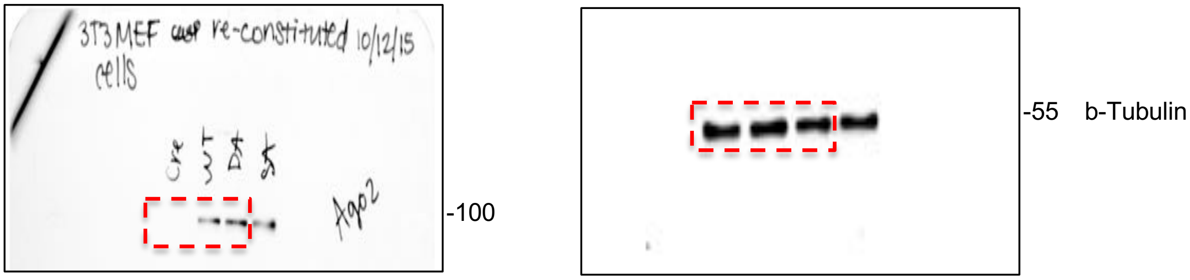

Supplementary Fig. 2b

Supplementary Fig. 9
(continued)

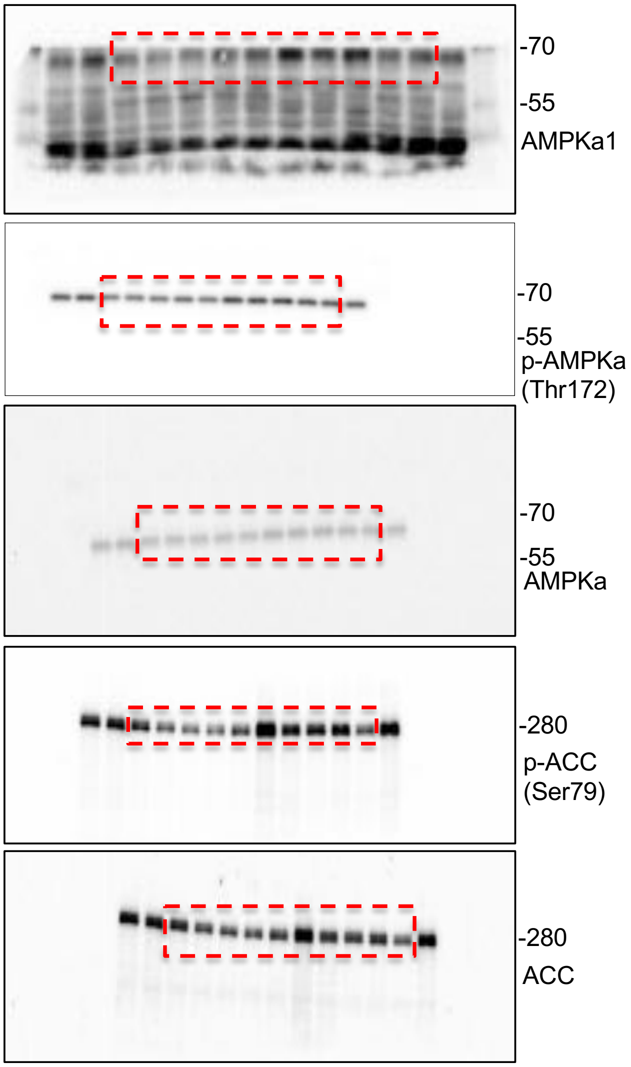

Supplementary Fig. 7a

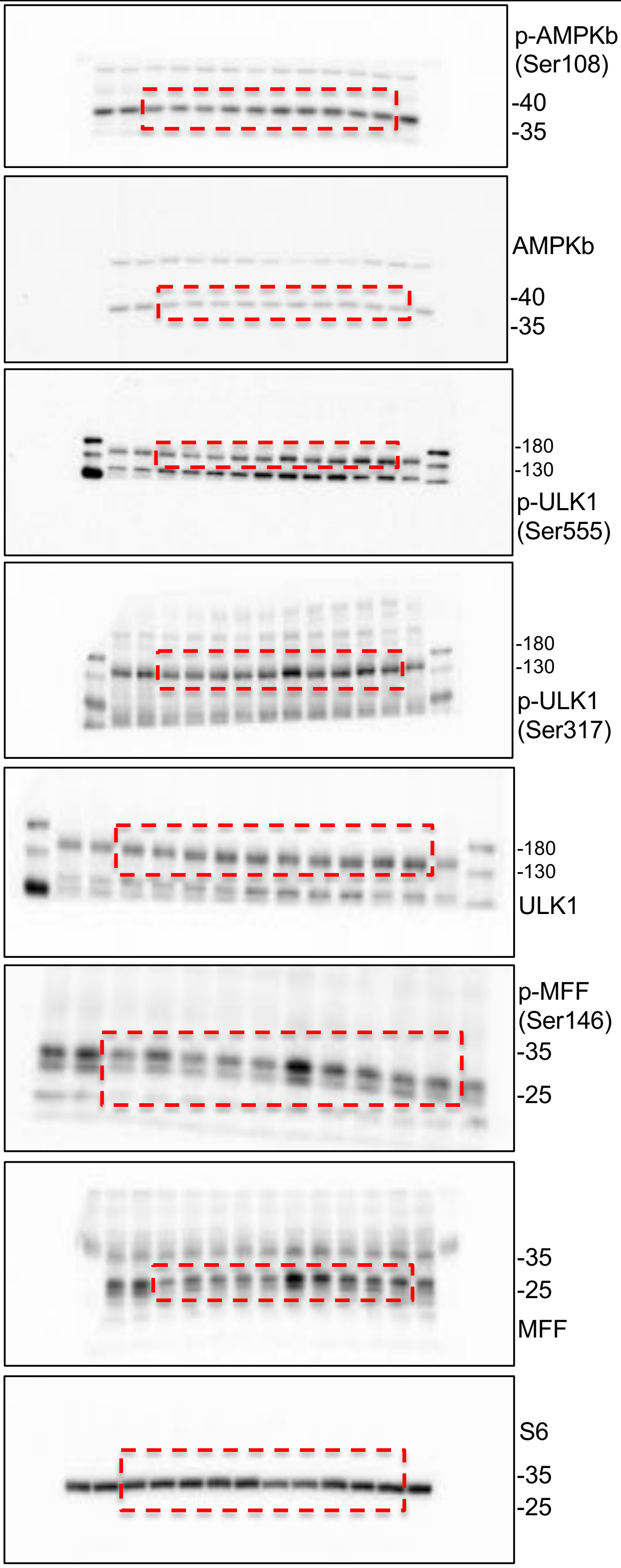

Supplementary Fig. 7a (continued)

Supplementary Fig. 9
(continued)

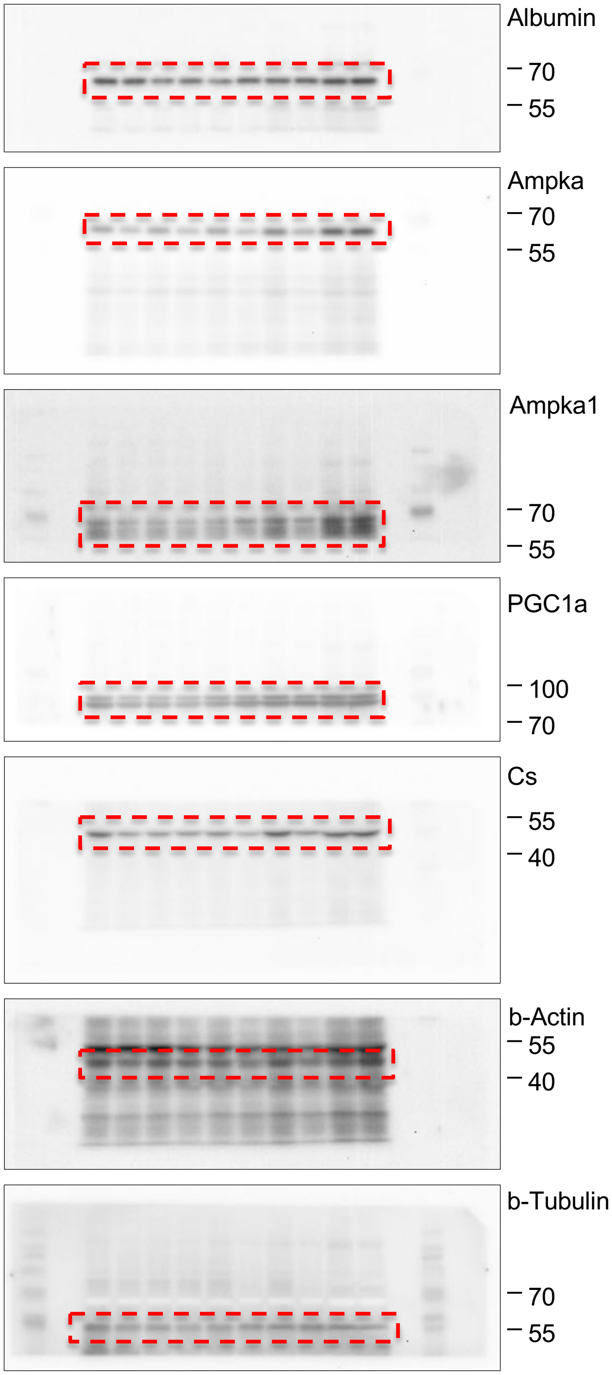

Supplementary Fig. 7c

Supplementary Fig. 9
(continued)

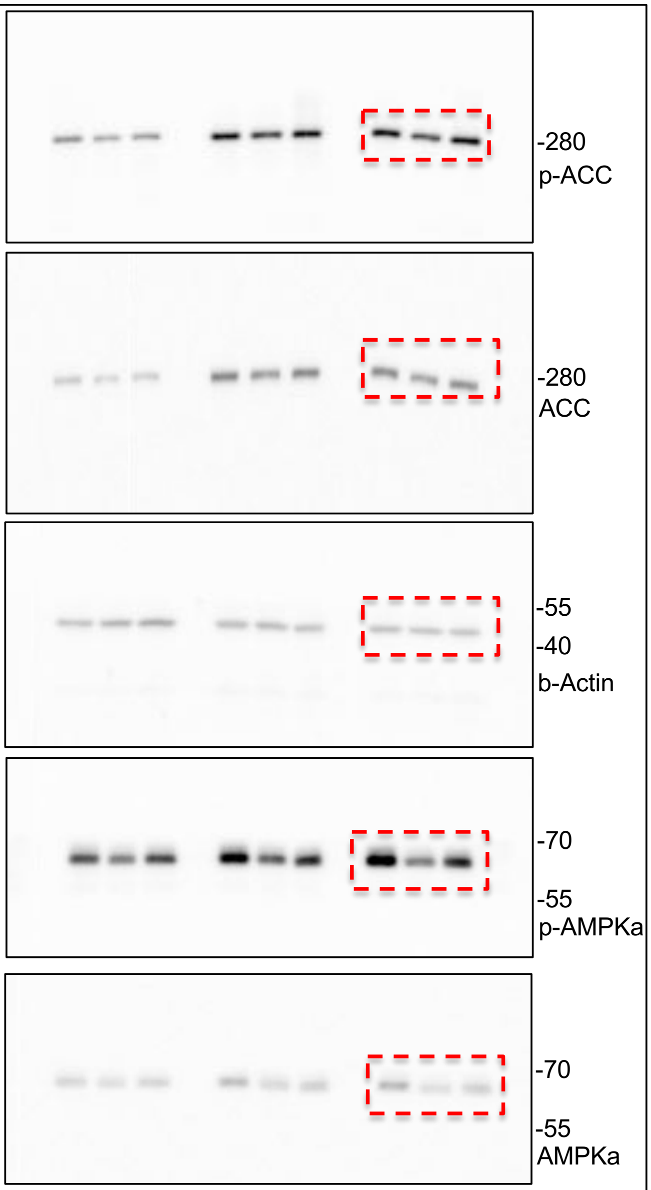

Supplementary Fig. 8a

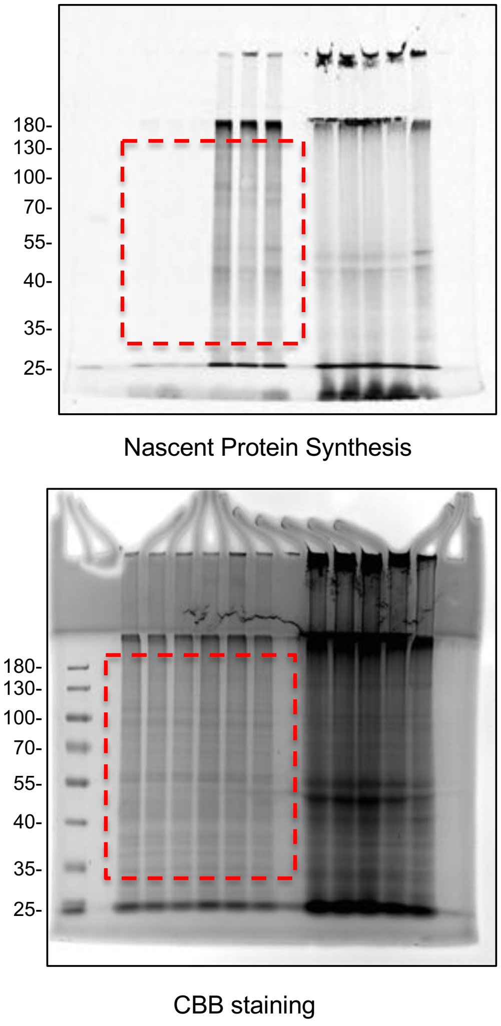

Supplementary Fig. 8b

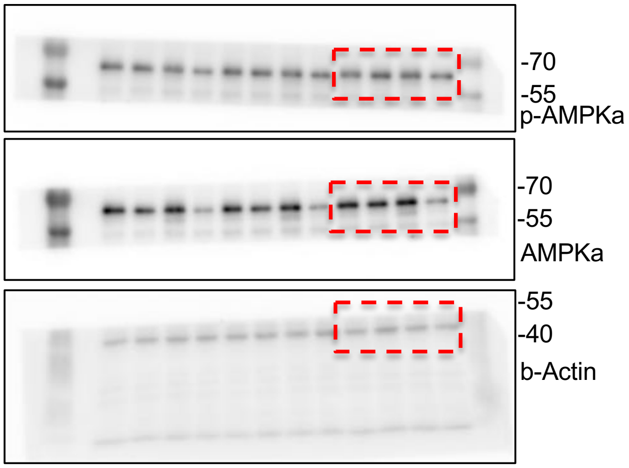

Supplementary Fig. 8c

Supplementary Table 1. Analysis of miRNA expression profile in the liver of L-Ago2 WT and KO mice.

	L-Ago2 WT	L-Ago2 KO	Relative Levels (WT/KO)
mmu-mir-22,mmu-miR-22-3p	474036.5679	473406.1337	1.001331698
mmu-mir-192,mmu-miR-192-5p	129635.1417	141111.9517	0.91866876
mmu-mir-21a,mmu-miR-21a-5p	97265.77427	93993.27762	1.034816284
mmu-mir-148a,mmu-miR-148a-3p	78595.54939	63169.6409	1.244198135
mmu-mir-26a-2,mmu-miR-26a-5p	78438.26342	81160.00777	0.966464464
mmu-mir-10a,mmu-miR-10a-5p	68232.23662	75835.38845	0.899741374
mmu-mir-27b,mmu-miR-27b-3p	64632.15311	48683.47854	1.327599322
mmu-mir-27a,mmu-miR-27a-3p	57994.79684	44232.24905	1.311142844
mmu-mir-122,mmu-miR-122-5p	38882.29391	39958.87866	0.973057683
mmu-mir-143,mmu-miR-143-3p	38097.75955	40414.97379	0.942664463
mmu-mir-30a,mmu-miR-30a-5p	38039.06879	41287.54381	0.921320701
mmu-let-7f-1,mmu-let-7f-5p	25461.72401	23591.93667	1.079255356
mmu-mir-191,mmu-miR-191-5p	18555.21117	16661.85803	1.113633974
mmu-mir-101b,mmu-miR-101b-3p	18008.58708	11683.03535	1.541430506
mmu-mir-30e,mmu-miR-30e-5p	16951.09138	23618.12037	0.717715513
mmu-mir-92a-1,mmu-miR-92a-3p	14996.62208	16532.51725	0.907098529
mmu-mir-194-1,mmu-miR-194-5p	14531.69849	9629.031272	1.509154771
mmu-mir-26b,mmu-miR-26b-5p	14250.8989	14264.58975	0.999040221
mmu-mir-30c-1,mmu-miR-30c-5p	14163.65048	15126.19512	0.936365714
mmu-mir-99b,mmu-miR-99b-5p	14058.27702	13852.75595	1.014836114
mmu-mir-29a,mmu-miR-29a-3p	12691.95895	12188.29459	1.041323613
mmu-mir-101a,mmu-miR-101a-3p	12241.94368	9802.275449	1.248887949
mmu-mir-92a-2,mmu-miR-92a-3p	12115.07217	14074.33941	0.860791531
mmu-mir-378a,mmu-miR-378a-3p	11223.01157	10844.70422	1.034884063
mmu-mir-125a,mmu-miR-125a-5p	10536.76946	10267.22245	1.026253157
mmu-let-7a-1,mmu-let-7a-5p	9438.46214	9124.685797	1.034387633
mmu-mir-100,mmu-miR-100-5p	8305.586844	9741.867417	0.852566196
mmu-mir-30d,mmu-miR-30d-5p	8199.385633	8145.013188	1.00667555
mmu-let-7g,mmu-let-7g-5p	8174.055739	7226.942108	1.131053164
mmu-mir-378c,mmu-miR-378c	6885.994957	6042.082268	1.139672492
mmu-mir-486a,mmu-miR-486a-5p	6570.978009	7344.09133	0.894729887
mmu-mir-486b,mmu-miR-486b-5p	6564.717711	7334.029687	0.895103782
mmu-mir-125b-1,mmu-miR-125b-5p	5977.867157	7248.404571	0.824714887
mmu-mir-151,mmu-miR-151-5p	5461.492675	5520.729226	0.989270158
mmu-mir-126a,mmu-miR-126a-3p	4998.664684	5809.684125	0.860402145
mmu-mir-30b,mmu-miR-30b-5p	4859.886395	4879.057619	0.996070712
mmu-let-7c-2,mmu-let-7c-5p	4655.984959	4685.04095	0.993798135
mmu-let-7c-1,mmu-let-7c-5p	4652.854701	4677.91073	0.994643757
mmu-mir-142a,mmu-miR-142a-5p	4275.795821	5352.665496	0.798816183
mmu-mir-99a,mmu-miR-99a-5p	3812.109952	3964.84009	0.961478866
mmu-mir-181a-1,mmu-miR-181a-5p	3423.459798	4359.551939	0.785277902
mmu-mir-151,mmu-miR-151-3p	3229.256872	3010.901942	1.072521435
mmu-mir-122,mmu-miR-122-3p	2566.777267	3335.200969	0.769601979
mmu-mir-107,mmu-miR-107-3p	2494.46579	1415.239536	1.762574976
mmu-mir-802,mmu-miR-802-5p	2399.389766	619.6246887	3.872327572
mmu-let-7i,mmu-let-7i-5p	2257.186113	2540.178047	0.888593662
mmu-let-7d,mmu-let-7d-5p	2243.348989	1940.969848	1.155787655
mmu-mir-103-1,mmu-miR-103-3p	2159.015525	1442.081419	1.497152308
mmu-mir-29c,mmu-miR-29c-3p	2111.767809	2287.713246	0.923091132
mmu-mir-203,mmu-miR-203-3p	2030.121628	1923.565571	1.055395074
mmu-mir-451a,mmu-miR-451a	2014.011122	2319.081238	0.868452165
mmu-mir-15a,mmu-miR-15a-5p	1611.519102	1916.634816	0.840806547
mmu-mir-199a-1,mmu-miR-199a-3p	1604.090444	1977.375903	0.811221802
mmu-mir-199b,mmu-miR-199b-3p	1604.090444	1977.375903	0.811221802
mmu-mir-93,mmu-miR-93-5p	1590.952897	1058.808363	1.502588148
mmu-mir-186,mmu-miR-186-5p	1578.965377	1320.10545	1.196090341
mmu-mir-140,mmu-miR-140-3p	1567.27108	1242.150041	1.261740554
mmu-mir-25,mmu-miR-25-3p	1555.206398	1573.332423	0.988479214
mmu-mir-340,mmu-miR-340-5p	1415.795299	1155.592951	1.225167822
mmu-mir-10b,mmu-miR-10b-5p	1400.13092	1385.240839	1.010749092
mmu-mir-193a,mmu-miR-193a-3p	1334.923053	1122.359081	1.189390344
mmu-mir-21a,mmu-miR-21a-3p	1174.560513	992.4980556	1.183438604
mmu-let-7b,mmu-let-7b-5p	1124.859631	1166.526038	0.964281632
mmu-mir-31,mmu-miR-31-5p	998.0168056	977.2368818	1.021263958
mmu-mir-181c,mmu-miR-181c-5p	858.9529755	874.616372	0.982091124
mmu-mir-423,mmu-miR-423-3p	848.9836023	820.5495387	1.034652464
mmu-mir-145a,mmu-miR-145a-3p	843.3584617	832.6542849	1.012855488
mmu-mir-23b,mmu-miR-23b-3p	804.3240749	722.047716	1.113948645
mmu-mir-16-1,mmu-miR-16-5p	799.047909	900.8268341	0.887016104
mmu-mir-351,mmu-miR-351-5p	781.102157	948.2732932	0.823709961
mmu-mir-126a,mmu-miR-126a-5p	748.1681075	871.255855	0.858723764
mmu-mir-130a,mmu-miR-130a-3p	722.5122801	416.4514339	1.734925663
mmu-mir-148a,mmu-miR-148a-5p	707.1439187	794.4648321	0.890088384
mmu-mir-199a-1,mmu-miR-199a-5p	699.5854413	855.6804444	0.817577924
mmu-mir-1948,mmu-miR-1948-3p	595.8536952	629.2797266	0.946882078
mmu-mir-148b,mmu-miR-148b-3p	552.4399224	463.3050087	1.192389273
mmu-mir-322,mmu-miR-322-5p	534.0884857	560.536907	0.952815914
mmu-mir-425,mmu-miR-425-5p	533.6559556	363.4629036	1.468254257
mmu-mir-19b-1,mmu-miR-19b-3p	526.4029598	550.6067525	0.956041599
mmu-mir-1843a,mmu-miR-1843a-5p	522.6939593	444.3091336	1.176419569

Top 15 miRNAs

Top 10 percent

Top 25 percent

mmu-mir-152,mmu-miR-152-3p	514.3601609	392.7480062	1.309644232
mmu-mir-200b,mmu-miR-200b-3p	492.3698629	773.8618144	0.636250366
mmu-mir-423,mmu-miR-423-5p	463.0762441	409.8138098	1.129967397
mmu-mir-1839,mmu-miR-1839-5p	461.4433452	315.2518564	1.463729192
mmu-mir-24-1,mmu-miR-24-3p	458.7903479	466.7211194	0.983007472
mmu-mir-24-2,mmu-miR-24-3p	458.7903479	466.7211194	0.983007472
mmu-mir-30a,mmu-miR-30a-3p	435.6409928	365.2459515	1.192733255
mmu-let-7d,mmu-let-7d-3p	435.0454082	409.2401586	1.063056494
mmu-mir-497a,mmu-miR-497a-5p	420.3902858	485.6119355	0.865691831
mmu-mir-98,mmu-miR-98-5p	378.9867304	339.006276	1.11793426
mmu-mir-501,mmu-miR-501-3p	377.4181186	346.2074791	1.090150102
mmu-mir-28a,mmu-miR-28a-5p	369.8826042	310.077243	1.192872462
mmu-mir-29b-1,mmu-miR-29b-3p	365.5471132	233.5968626	1.564863111
mmu-mir-335,mmu-miR-335-5p	341.9413915	369.8869297	0.92444843
mmu-mir-532,mmu-miR-532-5p	340.6996865	282.1620408	1.207461094
mmu-let-7e,mmu-let-7e-5p	300.8311071	273.112732	1.1014906
mmu-mir-1843b,mmu-miR-1843b-5p	290.6312604	253.1069751	1.148254647
mmu-mir-1948,mmu-miR-1948-5p	283.7495909	365.8921266	0.775500674
mmu-mir-24-2,mmu-miR-24-2-5p	275.4682212	345.5558383	0.797174264
mmu-mir-20a,mmu-miR-20a-5p	272.0381972	257.9374679	1.05466724
mmu-mir-127,mmu-miR-127-3p	267.8356669	353.9143642	0.756781001
mmu-mir-365-1,mmu-miR-365-3p	259.3805215	156.1557083	1.661037719
mmu-mir-30d,mmu-miR-30d-3p	258.7820452	260.4322451	0.993663612
mmu-mir-195a,mmu-miR-195a-5p	254.2853705	364.6171857	0.697403689
mmu-mir-342,mmu-miR-342-3p	253.5560098	277.9762637	0.912149859
mmu-mir-150,mmu-miR-150-5p	249.6881864	429.438016	0.581430095
mmu-mir-145a,mmu-miR-145a-5p	247.0929862	278.2538201	0.888012916
mmu-mir-146a,mmu-miR-146a-5p	246.4249991	333.8881993	0.738046447
mmu-mir-484,mmu-miR-484	243.3616824	184.1814325	1.321314961
mmu-mir-802,mmu-miR-802-3p	242.6579881	88.10097651	2.754316668
mmu-mir-30c-2,mmu-miR-30c-2-3p	236.5490168	293.594098	0.805700858
mmu-mir-17,mmu-miR-17-5p	234.3023811	146.4882123	1.599462356
mmu-mir-182,mmu-miR-182-5p	228.9455275	331.6040339	0.690418403
mmu-mir-142a,mmu-miR-142a-3p	225.3419101	274.9008011	0.81972082
mmu-mir-872,mmu-miR-872-5p	217.31051	297.4167467	0.730659966
mmu-let-7a-1,mmu-let-7a-1-3p	212.6502909	259.8205411	0.818450651
mmu-let-7c-2,mmu-let-7c-2-3p	212.6502909	259.8205411	0.818450651
mmu-mir-133a-1,mmu-miR-133a-3p	207.9770553	143.471225	1.449608137
mmu-mir-378a,mmu-miR-378a-5p	204.3536872	157.3706126	1.298550497
mmu-mir-144,mmu-miR-144-3p	203.1147966	254.1173334	0.799295325
mmu-mir-652,mmu-miR-652-3p	198.1084679	185.9215303	1.065548824
mmu-mir-141,mmu-miR-141-3p	189.6815406	111.6023477	1.699619627
mmu-mir-200a,mmu-miR-200a-3p	184.9898596	214.1813296	0.863706748
mmu-mir-744,mmu-miR-744-5p	179.5654773	143.9408919	1.247494543
mmu-mir-872,mmu-miR-872-3p	162.6274076	163.8441067	0.992574044
mmu-mir-22,mmu-miR-22-5p	159.1328612	272.8545908	0.583214894
mmu-mir-411,mmu-miR-411-5p	158.9576813	255.3350902	0.622545382
mmu-mir-338,mmu-miR-338-3p	158.4027609	176.2440339	0.898769492
mmu-mir-378b,mmu-miR-378b	156.0852464	143.9478716	1.084317848
mmu-mir-320,mmu-miR-320-3p	155.3105661	84.32179909	1.841879179
mmu-mir-328,mmu-miR-328-3p	151.0763071	121.6179408	1.242220565
mmu-mir-30e,mmu-miR-30e-3p	143.9269525	136.7651898	1.052365392
mmu-mir-28a,mmu-miR-28a-3p	140.887681	153.1212918	0.920105097
mmu-mir-455,mmu-miR-455-5p	138.91057	132.6023522	1.047572443
mmu-let-7f-2,mmu-let-7f-2-3p	137.2326278	174.7560288	0.785281222
mmu-mir-221,mmu-miR-221-3p	133.4532069	99.16596352	1.345756167
mmu-mir-450a-1,mmu-miR-450a-5p	131.1431044	144.855184	0.905339393
mmu-mir-139,mmu-miR-139-5p	125.4123814	146.7557956	0.854565102
mmu-mir-106b,mmu-miR-106b-5p	120.4915732	105.9880684	1.13684092
mmu-mir-322,mmu-miR-322-3p	119.410744	122.0990774	0.977982361
mmu-mir-1247,mmu-miR-1247-5p	119.2721038	97.92157464	1.218037028
mmu-mir-185,mmu-miR-185-5p	110.4074302	63.47780109	1.739307732
mmu-mir-192,mmu-miR-192-3p	109.0277086	112.4280916	0.969755041
mmu-mir-455,mmu-miR-455-3p	108.3962564	92.14777174	1.176330738
mmu-mir-361,mmu-miR-361-3p	108.1124374	89.94055123	1.202043304
mmu-mir-152,mmu-miR-152-5p	105.8941898	77.89880046	1.35938152
mmu-mir-181b-1,mmu-miR-181b-5p	101.4557126	125.4616349	0.808659258
mmu-mir-92a-1,mmu-miR-92a-1-5p	94.44869089	144.8896924	0.651866184
mmu-mir-339,mmu-miR-339-5p	90.26563976	79.5628127	1.134520471
mmu-let-7b,mmu-let-7b-3p	90.00369215	141.5001101	0.636068001
mmu-mir-676,mmu-miR-676-3p	85.43489138	98.14311526	0.870513343
mmu-mir-210,mmu-miR-210-3p	84.54540256	86.44247454	0.978053937
mmu-mir-32,mmu-miR-32-5p	81.73547658	70.77759733	1.154821295
mmu-mir-194-2,mmu-miR-194-2-3p	80.38004029	87.79038123	0.915590514
mmu-mir-326,mmu-miR-326-3p	78.1934948	81.92456024	0.954457303
mmu-mir-222,mmu-miR-222-3p	78.16179273	48.90224742	1.598327211
mmu-mir-23a,mmu-miR-23a-3p	78.05814672	97.3408792	0.801905092
mmu-mir-128-1,mmu-miR-128-3p	75.40215285	63.82891876	1.181316468
mmu-mir-149,mmu-miR-149-5p	74.26625804	53.38414667	1.391166904
mmu-mir-101a,mmu-miR-101a-5p	73.4622365	35.55709867	2.066035735
mmu-mir-128-2,mmu-miR-128-3p	72.29026054	63.07868485	1.146033097
mmu-mir-671,mmu-miR-671-3p	70.74568437	72.70368257	0.973068789
mmu-mir-5099,mmu-miR-5099	67.37773754	93.04349801	0.7241531
mmu-mir-19a,mmu-miR-19a-3p	66.34219658	70.29592964	0.943755875

mmu-mir-214,mmu-miR-214-3p	65.41663142	85.28508995	0.767034794
mmu-mir-434,mmu-miR-434-3p	64.1213119	85.90578405	0.746414373
mmu-mir-1198,mmu-miR-1198-5p	64.02008947	61.18259065	1.046377553
mmu-mir-136,mmu-miR-136-3p	59.40056591	77.00500554	0.771385775
mmu-mir-148b,mmu-miR-148b-5p	59.33191412	42.80585844	1.386069951
mmu-mir-339,mmu-miR-339-3p	58.82529768	50.49573625	1.164955738
mmu-mir-1981,mmu-miR-1981-3p	58.20126216	25.12083022	2.316852654
mmu-mir-146b,mmu-miR-146b-5p	57.05485025	63.34478871	0.900703142
mmu-mir-345,mmu-miR-345-5p	56.26441482	47.05117566	1.195813155
mmu-mir-511,mmu-miR-511-3p	56.05297166	65.54794381	0.855144622
mmu-let-7f-1,mmu-let-7f-1-3p	52.79021624	62.01184163	0.851292509
mmu-mir-106b,mmu-miR-106b-3p	52.5011544	42.81536231	1.226222355
mmu-mir-33,mmu-miR-33-5p	51.61188392	33.80054461	1.526954211
mmu-mir-421,mmu-miR-421-3p	48.255973	42.29969999	1.140811235
mmu-mir-136,mmu-miR-136-5p	45.26739286	45.8943148	0.986339878
mmu-mir-26b,mmu-miR-26b-3p	45.03649709	66.96539096	0.672533923
mmu-mir-340,mmu-miR-340-3p	44.85415026	35.08845196	1.27831659
mmu-mir-125b-2,mmu-miR-125b-2-3p	42.74544181	44.12176366	0.968806282
mmu-mir-187,mmu-miR-187-3p	42.65644224	45.67474737	0.93391742
mmu-mir-664,mmu-miR-664-3p	42.34792778	37.85481601	1.118693267
mmu-mir-221,mmu-miR-221-5p	42.01075368	30.13089903	1.394274815
mmu-mir-99a,mmu-miR-99a-3p	41.57213399	66.55826755	0.624597597
mmu-mir-132,mmu-miR-132-3p	41.47156179	44.28281845	0.93651586
mmu-mir-361,mmu-miR-361-5p	39.9009583	31.27825602	1.275677208
mmu-mir-362,mmu-miR-362-3p	39.5937491	38.97567181	1.015858028
mmu-mir-374b,mmu-miR-374b-5p	38.11651945	38.28294033	0.995652871
mmu-mir-98,mmu-miR-98-3p	35.11767194	30.07838314	1.167538553
mmu-mir-96,mmu-miR-96-5p	35.01774997	40.10151415	0.873227625
mmu-mir-34a,mmu-miR-34a-5p	34.98713003	43.68413547	0.800911582
mmu-mir-429,mmu-miR-429-3p	34.24611212	36.17128228	0.946776281
mmu-mir-874,mmu-miR-874-3p	33.96601717	29.76828433	1.141013597
mmu-mir-1249,mmu-miR-1249-3p	32.38688347	44.23480262	0.732158426
mmu-mir-676,mmu-miR-676-5p	31.95068256	25.50844942	1.25255291
mmu-mir-212,mmu-miR-212-5p	30.87140363	37.38567283	0.825754929
mmu-mir-381,mmu-miR-381-3p	29.51246163	42.19064734	0.699502461
mmu-mir-7a-1,mmu-miR-7a-5p	28.70781645	18.5417906	1.548276381
mmu-mir-7a-2,mmu-miR-7a-5p	28.70781645	18.5417906	1.548276381
mmu-mir-223,mmu-miR-223-3p	28.45986026	30.24292779	0.941041835
mmu-mir-664,mmu-miR-664-5p	28.21146737	33.87708205	0.832759661
mmu-mir-744,mmu-miR-744-3p	28.03982461	23.17472703	1.209931171

# the 3rd Quartile (top 25%) abundant miRs (221 / 881 in total) *Redundant miRNAs are removed from this list.

Supplementary Table 2. Ortholog mapping of miRNAs.

Mouse microRNA	miRbase mapping relationship	mirortho mapping relationship	Human microRNA(s)
mmu-miR-101a-3p	no ortholog	1-to-1	miR-101a
mmu-miR-101a-5p	no ortholog	1-to-1	miR-101a
mmu-miR-101b-3p	no ortholog	1-to-1	miR-101b-3p
mmu-miR-103-3p	no ortholog	1-to-many	niR-103-3p_1, miR-103-3p_2
mmu-miR-107-3p	no ortholog	no ortholog	n/a
mmu-miR-130a-3p	1-to-1	1-to-1	miR-130a-3p
mmu-miR-140-3p	1-to-1	1-to-1	miR-140-3p
mmu-miR-141-3p	1-to-1	no ortholog	miR-141-3p
mmu-miR-148a-3p	1-to-1	1-to-1	miR-148a-3p
mmu-miR-148b-3p	1-to-1	1-to-1	miR-148b-3p
mmu-miR-148b-5p	1-to-1	1-to-1	miR-148b-5p
mmu-miR-152-3p	1-to-1	no ortholog	miR-152-3p
mmu-miR-17-5p	1-to-1	1-to-1	miR-17-5p
mmu-miR-1839-5p	no ortholog	1-to-many	R-1839-5p_1, miR-1839-5p_2
mmu-miR-185-5p	1-to-1	1-to-1	miR-185-5p
mmu-miR-194-5p	1-to-many	1-to-many	niR-194-5p_1, miR-194-5p_2
mmu-miR-1981-3p	no ortholog	no ortholog	n/a
mmu-miR-27a-3p	1-to-1	1-to-1	miR-27a-3p
mmu-miR-27b-3p	1-to-1	1-to-1	miR-27b-3p
mmu-miR-29b-3p	1-to-many	1-to-many	niR-29b-3p_1, miR-29b-3p_2
mmu-miR-320-3p	no ortholog	1-to-1	miR-320-3p
mmu-miR-365-3p	no ortholog	1-to-many	niR-365-3p_1, miR-365-3p_2
mmu-miR-425-5p	1-to-1	1-to-1	miR-425-5p
mmu-miR-802-3p	no ortholog	1-to-1	miR-802-3p
mmu-miR-802-5p	no ortholog	1-to-1	miR-802-5p
mmu-miR-93-5p	1-to-1	no ortholog	miR-93-5p

Supplementary Table 3. GWAS signal near miRNAs

Human microRNA	Phenotype:Ancestry	SNP rsid	Distance to SNP	miR chr	miR start	miR end	SNP chr	SNP start	SNP end
miR-148a-3p	Body_mass_index:AA	rs6953596	245	chr7	25989542	25989563	chr7	25989298	25989298
miR-101b-3p	Mean_corpuscular_hemoglobin:AS	rs17803780	639	chr9	4850285	4850381	chr9	4849647	4849647
miR-101b-3p	Red_blood_cell_traits:EU	rs17803780	639	chr9	4850285	4850381	chr9	4849647	4849647
miR-101b-3p	Mean_corpuscular_hemoglobin:EU	rs17803780	639	chr9	4850285	4850381	chr9	4849647	4849647
miR-101b-3p	Mean_corpuscular_volume:EU	rs17803780	639	chr9	4850285	4850381	chr9	4849647	4849647
miR-101b-3p	Mean_corpuscular_volume:AS	rs17803780	639	chr9	4850285	4850381	chr9	4849647	4849647
miR-17-5p	Height:EU	rs4284505	1401	chr13	92002872	92002894	chr13	92001472	92001472
miR-148a-3p	Triglycerides:EU	rs4722551	2263	chr7	25989542	25989563	chr7	25991826	25991826
miR-148a-3p	LDL_cholesterol:EU	rs4722551	2263	chr7	25989542	25989563	chr7	25991826	25991826
miR-148a-3p	Cholesterol_total:EU	rs4722551	2263	chr7	25989542	25989563	chr7	25991826	25991826
miR-320-3p	Chronic_lymphocytic_leukemia:EU	rs117579506	3378	chr19	47212536	47212617	chr19	47209159	47209159
miR-320-3p	Type_1_diabetes:EU	rs425105	4056	chr19	47212536	47212617	chr19	47208481	47208481
miR-148b-3p	Platelet_counts:EU	rs4326844	5387	chr12	54731062	54731083	chr12	54736469	54736470
miR-148b-3p	Mean_platelet_volume:EU	rs4326844	5387	chr12	54731062	54731083	chr12	54736470	54736470
miR-148b-5p	Platelet_counts:EU	rs4326844	5425	chr12	54731024	54731045	chr12	54736469	54736470
miR-148b-5p	Mean_platelet_volume:EU	rs4326844	5425	chr12	54731024	54731045	chr12	54736470	54736470
miR-365-3p_2	Height:EU	rs246185	7711	chr16	14403142	14403228	chr16	14395432	14395432
miR-365-3p_2	Menarche_age_at_onset:EU	rs246185	7711	chr16	14403142	14403228	chr16	14395432	14395432
miR-29b-3p_2	Heart_rate:EU	rs12568382	9403	chr1	207975795	207975817	chr1	207966393	207966393

Supplementary Table 4. List of primers used in this study

Gene	Species	Accession #	Forward	Reverse
**mRNA**				
β-actin	Mouse	NM_007393	5'- GGC TGT ATT CCC CTC CAT CG -3'	5'- CCA GTT GGT AAC AAT GCC ATG T -3'
G6Pase	Mouse	NM_008061	5'- CGA CTC GCT ATC TCC AAG TGA -3'	5'- GTT GAA CCA GTC TCC GAC CA -3'
Ppar-γ	Mouse	NM_011146.3	5'- GCA TGG TGC CTT CGC TGA -3'	5'- TGG CAT CTC TGT GTC AAC CAT G -3'
Ppar-α	Mouse	NM_001113418.1	5'- TGT TTG TGG CTG CTA TAA TTT GC -3'	5'- GCA ACT TCT CAA TGT AGC CTA TGT TT -3'
Tfam	Mouse	NM_009360.4	5'- CAC CCA GAT GCA AAA CTT TCA G -3'	5'- CTG CTC TTT ATA CTT GCT CAC AG -3'
Cs	Mouse	NM_026444.3	5'- GGG ACT TGT GTA TGA GAC TTC G -3'	5'- AGC CAA AAT AAG CCC TCA GG -3'
Cytc	Mouse	NM_007808.4	5'- GGA GGC AAG CAT AAG ACT GG -3'	5'- TCC ATC AGG GTA TCC TCT CC -3'
mt-Cox1	Mouse	NC_005089.1 (5328-6872)	5'- TCC AAC TCA TCC CTT GAC ATC -3'	5'- TCC TGC TAT GAT AGC AAA CAC T -3'
mt-Cox2	Mouse	NC_005089.1 (7013-7696)	5'- CTA ATT AGC TCC TTA GTC CTC -3'	5'- TTC GTA GCT TCA GTA TCA TTG -3'
mt-Cox3	Mouse	NC_005089.1 (8607-9390)	5'- ATT CTA TTC ATC GTC TCG GAA -3'	5'- AAG GCT ATG ATG AGC TCA TGT -3'
mt-Atp6	Mouse	NC_005089.1 (7927-8607)	5'- TAA TCA ACA ACC GTC TCC ATT C -3'	5'- GTG TCG GAA GCC TGT AAT TAC -3'
mt-Atp8	Mouse	NC_005089.1 (7766-7969)	5'- TGC CAC AAC TAG ATA CAT CAA -3'	5'- GGT AAT GAA TGA GGC AAA TAG -3'
mt-Cytb	Mouse	NC_005089.1 (14145-15288)	5'- GCA ACG AAG CCT AAT ATT CC -3'	5'- TGA GAT TGG TAT AAG AAT TAA -3'
mt-Nd1	Mouse	NC_005089.1 (2751-3707)	5'- TTA CCA GAA CTC TAC TCA ACT -3'	5'- ATC GTA ACG GAA GCG TGG ATA -3'
mt-Nd2	Mouse	NC_005089.1 (3914-4951)	5'- TCA ATA ATT ATC CTC CTG GCC -3'	5'- ATG ATA GTA GAG TTG AGT AGC -3'
mt-Nd3	Mouse	NC_005089.1 (9459-9806)	5'- TTC TAG TTG CAT TCT GAC TCC -3'	5'- ATA GAA TTG TGA CTA GAA TAA -3'
mt-Nd4	Mouse	NC_005089.1 (10167-11544)	5'- GCC TGA TTA CTG CCA CTA ATA -3'	5'- GGT TCC CTC ATC GGG TAA TAA -3'
mt-Nd4L	Mouse	NC_005089.1 (9877-10173)	5'- ACT ATC ACT TCT AGG GAC ACT -3'	5'- TTG GAC GTA ATC TGT TCC GT -3'
mt-Nd5	Mouse	NC_005089.1 (11742-13565)	5'- AAC CAC ACC TAG CAT TCC TAC -3'	5'- CAG GCG TTG GTG TTG CAG GTA -3'
mt-Nd6	Mouse	NC_005089.1 (13552-14070, complement)	5'- ACA ACT ATA TAT TGC CGC TAC -3'	5'- GAT ATA CGA CTG CTA TAG CTA -3'
Oct1	Mouse	NM_009202.5	5'- GAC GCC TGG AAA GTG GAC C -3'	5'- GCA ACA TGG ATG TAT AGT CTG GG -3'
Oct3	Mouse	NM_011395	5'- AGC CAG CCC GAC TAC TAT TGG T -3'	5'- TGA GCT CTG AGC TGG TAT TAG T -3'
Gpd2	Mouse	NM_001145820	5'- GAA GGG GAC TAT TCT TGT GGG T -3'	5'- GGA TGT CAA ATT CGG GTG TGT -3'
Cav-1	Mouse	AB029929	5'- ATG TCT GGG GGC AAA TAC GTG -3'	5'- CGC GTC ATA CAC TTG CTT CT -3'
Hnf1β	Mouse	NM_009330.3	5'- CAC CAA GCC GGT TTT CCA TAC -3'	5'- GGA GTG TCA TAG TCG TCG CC -3'
Pgc1a	Mouse	NM_008904	5'- CAG CCT CTT TGC CCA GAT CT -3'	5'- CCG CTA GCA AGT TTG CCT CA -3'
Tnfa	Mouse	NM_013693.3	5'- GCT ACG ACG TGG GCT ACA G -3'	5'- CCC TCA CAC TCA GAT CAT CTT CT -3'
Srebp1c	Mouse	NM_011480.4	5'- GGA GCC ATG GAT TGC ACA TT -3'	5'- GCT TCC AGA GAG GAG GCC AG -3'
Scd1	Mouse	NM_009127.4	5'- CGG GAT TGA ATG TTC TTG TCG T -3'	5'- TTC TTG CGA TAC ACT CTG GTG C -3'
Fasn	Mouse	NM_007988.3	5'- AAG GCT GGG CTC TAT GGA TT -3'	5'- GGA GTG AGG CTG GGT TGA TA -3'
Ampka1	Mouse	NM_001013367.3	5'- TGT TCC AGC AGA TCC TTT CC -3'	5'- ATA ATT GGG TGA GCC ACA GC -3'
Ampka2	Mouse	XM_006502651.3	5'- GGG TGA AGA TCG GAC ACT ACG T -3'	5'- GTG TTC TCC AAT CTT CAC TTT G -3'
Ampkb1	Mouse	NM_031869.2	5'- CAT CCT CCC GCC ACA CCT GC -3'	5'- GAG CAC CAT CAC TCC ATC CT -3'
Ampkb2	Mouse	NM_182997.2	5'- GGG AAA GGA GCA CAA GAT C -3'	5'- CTG CTG CCA GGG TAC AAA C -3'
Idh3b	Mouse	NM_130884.4	5'- ATC TGA GCG AGG TGC AGA AT -3'	5'- TAC GTT GGC AAA CAA ATC CA -3'
Fh1	Mouse	NM_010209.2	5'- AGC AAT GCA TAT TGC TGC TG -3'	5'- CGC ATA CTG GAC TTG CTG AA -3'
Mdh1	Mouse	NM_001316675.1	5'- GAA GCC CTG AAA GAC GAC AG -3'	5'- TCG ACA CGA ACT CTC CCT CT -3'
Cpt1a	Mouse	NM_013495.2	5'- GCT GGG CTA CTC AGA GGA TG -3'	5'- CAC TGT AGC CTG GTG GGT TT -3'
Pdhb	Mouse	NM_024221.3	5'- TCG AAG CCA TAG AAG CCA GT -3'	5'- AGG CAT AGG GAC ATC AGC AC -3'
Acss1	Mouse	NM_080575.2	5'- ACC AGA TCC TGG TGG TGA AG -3'	5'- TCC TCC AGG GTA GTG GTG TC -3'
Acss2	Mouse	NM_019811.3	5'- GCT TCT TTC CCA TTC TTC GGT -3'	5'- CCC GGA CTC ATT CAG GAT TG -3'
Acly	Mouse	NM_001199296.1	5'- GAT GAA GTG GCA CCT GCA AAG -3'	5'- GGT ATG TCG GCT GAA GAG GGT -3'
Sod1	Mouse	NM_011434.1	5'- CCA GTG CAG GAC CTC ATT TT -3'	5'- TTG TTT CTC ATG GAC CAC CA -3'
Sod2	Mouse	NM_013671.3	5'- CCG AGG AGA AGT ACC ACG AG -3'	5'- GCT TGA TAG CCT CCA GCA AC -3'
Il1b	Mouse	NM_008361.4	5'- GCA ACT GTT CCT GAA CTC AAC T -3'	5'- ATC TTT TGG GGT CGC TCA ACT -3'
Atf4	Mouse	NM_009716.3	5'- CCT TCG ACC AGT CGG GTT TG -3'	5'- CTG TCC CGG AAA AGG CAT CC -3'
mtDNA	Mouse	NC_005089.1	5'- CCC AGC TAC TAC CAT CAT TCA AGT -3'	5'- GAT GGT TTG GGA GAT TGG TTG ATG -3'
5S rRNA	Mouse	NR_030686.1	5'- GGC CAT ACC ACC CTG AAC GC -3'	5'- CAG CAC CCG GTATTCCCAGG -3'
12S rRNA	Mouse	NC_005089.1	5'- CAA ACT GGG ATT AGA TAC CCC ACT AT -3'	5'- GAG GGT GAC GGG CGG TGT GT -3'
**miRNA**				
miR-802	Mouse	MIMAT0004188	UCA GUA ACA AAG AUU CAU CCU U	
miR-107	Mouse	MIMAT0000647	AGC AGC AUU GUA CAG GGC UAU CA	
miR-320	Mouse	MIMAT0000666	AAA AGC UGG GUU GAG AGG GCG A	
miR-29b	Mouse	MIMAT0000127	UAG CAC CAU UUG AAA UCA GUG UU	
miR-33	Mouse	MIMAT0004666	CAA UGU UUC CAC AGU GCA UCA C	
miR-93	Mouse	MIMAT0004636	ACU GCU GAG CUA GCA CUU CCC G	
miR-103	Mouse	MIMAT0000546	AGC AGC AUU GUA CAG GGC UAU GA	
miR-130a	Mouse	MIMAT0000141	CAG UGC AAU GUU AAA AGG GCA U	
miR-27b	Mouse	MIMAT0000126	UUC ACA GUG GCU AAG UUC UGC	
miR-152	Mouse	MIMAT0000162	UCA GUG CAU GAC AGA ACU UGG	
miR-148a	Mouse	MIMAT0000516	UCA GUG CAC UAC AGA ACU UUG U	
miR-148b	Mouse	MIMAT0000580	UCA GUG CAU CAC AGA ACU UUG U	
snoRNA202	Mouse	AF357327	GCT GTA CTG ACT TGA TGA AAG TAC TTT TGA ACC CTT TTC CAT CTG ATG	
**pri-miR**				
pri-mir-802	Mouse	MI0004249	UCA GUA ACA AAG AUU CAU CCU U	
pri-mir-107	Mouse	MI0000684	AGC AGC AUU GUA CAG GGC UAU CA	
pri-mir-103-1	Mouse	MI0000587	AGC AGC AUU GUA CAG GGC UAU GA	
pri-mir-103-2	Mouse	MI0000588	AGC AGC AUU GUA CAG GGC UAU GA	
pri-mir-130a	Mouse	MI0000156	GCU CUU UUC ACA UUG UGC UAC U	
pri-miR-152	Mouse	MI0000174	UAG GUU CUG UGA UAC ACU CCG ACU	
pri-miR-148a	Mouse	MI0000550	UCA GUG CAC UAC AGA ACU UUG U	
pri-miR-148b	Mouse	MI0000617	GAA GUU CUG UUA UAC ACU CAG GCU	
